# Natural Killer–Dendritic Cell Interactions in Liver Cancer: Implications for Immunotherapy

**DOI:** 10.3390/cancers13092184

**Published:** 2021-05-01

**Authors:** Valentina Cazzetta, Sara Franzese, Claudia Carenza, Silvia Della Bella, Joanna Mikulak, Domenico Mavilio

**Affiliations:** 1 Laboratory of Clinical and Experimental Immunology, IRCCS Humanitas Research Hospital, 20089 Rozzano, Italy; Valentina.Cazzetta@humanitasresearch.it (V.C.); sara.franzese@humanitasresearch.it (S.F.); Claudia.Carenza@humanitasresearch.it (C.C.); silvia.dellabella@unimi.it (S.D.B.); joanna.mikulak@humanitasresearch.it (J.M.); 2 Department of Medical Biotechnologies and Translational Medicine, University of Milan, 20122 Milan, Italy

**Keywords:** innate immunity, DCs–NK cell interaction, liver cancer, cancer therapy

## Abstract

**Simple Summary:**

The reciprocal crosstalk between dendritic cells (DCs) and natural killer (NK) cells plays a pivotal role in regulating immune defense against viruses and tumors. The Th-cell polarizing ability, cytokine-producing capacity, chemokine expression, and migration of DCs are regulated by activated NK cells. Conversely, the effector functions including lysis and cytokine production, proliferation, and migration of NK cells are influenced by close interactions with activated DCs. In this review, we explore the impact of DC–NK cell crosstalk and its therapeutic potential in immune control of liver malignances.

**Abstract:**

Natural killer (NK) and dendritic cells (DCs) are innate immune cells that play a crucial role in anti-tumor immunity. NK cells kill tumor cells through direct cytotoxicity and cytokine secretion. DCs are needed for the activation of adaptive immune responses against tumor cells. Both NK cells and DCs are subdivided in several subsets endowed with specialized effector functions. Crosstalk between NK cells and DCs leads to the reciprocal control of their activation and polarization of immune responses. In this review, we describe the role of NK cells and DCs in liver cancer, focusing on the mechanisms involved in their reciprocal control and activation. In this context, intrahepatic NK cells and DCs present unique immunological features, due to the constant exposure to non-self-circulating antigens. These interactions might play a fundamental role in the pathology of primary liver cancer, namely hepatocellular carcinoma (HCC) and intrahepatic cholangiocarcinoma (ICC). Additionally, the implications of these immune changes are relevant from the perspective of improving the cancer immunotherapy strategies in HCC and ICC patients.

## 1. Introduction

The liver represents an important immunological organ in which a unique microenvironment shapes both the innate and adaptive immune responses to maintain a correct balance between immune tolerance and immune activation. About 80% of liver blood flow derives from the gastrointestinal tract, via the portal vein, carrying a high load of bacterial antigens. Accordingly, liver tissue is highly enriched in cells belonging to both the innate and adaptive immune systems [1]. The intimate interactions among different liver-resident immune cells, facilitated by low-pressure blood flow, the fenestrated endothelium, and lack of a basement membrane, are crucial for preventing liver injury. Their dysregulation plays a critical role in the pathogenesis of several hepatic diseases, such as viral hepatitis, autoimmune disorders, and tumors. 

Cells of the innate immune system orchestrate the first line of the immune response to pathogenic stimulus, or damage to host cells and tissues. DCs and NK cells are specialized sensors of the innate immune system with complementary and partially overlapping functions that play a critical role in the defense against cancer and infections. The outcome of NK–DC interaction leads to both NK cell and DC activation, which influences the innate and adaptive immune responses via cell-to-cell interactions and through secretion of numerous soluble factors, including chemokines and cytokines.

This review addresses different mechanisms of the NK–DC crosstalk in the context of liver homeostasis and regeneration, as well as neoplastic transformation, referring to hepatocellular carcinoma (HCC) and intrahepatic cholangiocarcinoma (ICC) microenvironments. Finally, we discuss the potential clinical implications in this field.

## 2. Implication of NK–DC Crosstalk in Cancer Immunity

### 2.1. NK Cells in Cancer Immunity

NK cells are large granular lymphocytes of the innate immune system. They circulate throughout the body and are present in both lymphoid organs and non-lymphoid peripheral tissues. NK cells are involved in direct innate immune reactions against pathogens and malignant cells. Importantly, NK cells link the innate and adaptive immune responses [2,3]. Human peripheral blood NK cells represent about 10% of circulating lymphocytes and are subdivided into two main subsets, based on the differential expression of CD56 and CD16 markers—CD56^bright^ (CD56^bright^/CD16^−^) and CD56^dim^ (CD56^dim^/CD16^+^) NK cells. The dominant mechanism regulating NK cell activity ensures immune-tolerance toward autologous cells, by engaging several inhibitory NK receptors (iNKRs) that are spared of NK cell cytotoxicity [4]. The iNKRs include inhibitory killer immunoglobulin-like receptors (iKIRs), and the C-type lectin like receptor NKG2A, which bind several alleles of the major histocompatibility complex class I (MHC-I) and a non-classical MHC-I complex, HLA-E, respectively. The absence of MHC-I on target cells licenses NK cell killing via the engagement of activating NK receptors (aNKRs), which bind their putative ligands expressed on infected or tumor-transformed cells [5]. aNKRs are natural cytotoxicity receptors (NCRs) NKp30, NKp46, and NKp44, and the C-type lectin receptors NKG2D and NKG2C. The NK CD56^bright^ and CD56^dim^ cell subsets have distinct roles in immunity. The CD56^bright^ cell subset serves more as an immune-modulator and the second CD56^dim^ population acts mainly as cytotoxic effectors [6]. NK CD56^dim^ cells, which represent the largest population in the blood (up to 90%), exert high Interferon (IFN)-γ production, cellular-mediated cytotoxicity, and antibody-dependent cellular cytotoxicity (ADCC) in response to the stressed target cells and several interleukins (ILs) (i.e., IL-12, IL-15, and IL-18). The poor cytotoxic CD56^bright^ NK cells, also unable to perform ADCC, have important regulatory functions through the secretion of chemokines and pro-inflammatory cytokines (i.e., IFN-γ and tumor necrosis factor (TNF)-α) in response to different stimuli (i.e., IL-1β, IL-2, IL-12, IL-15, and IL-18) delivered by the surrounding cells at tissue sites (i.e., macrophages, DCs, and T lymphocytes) [2,7,8].

Other than classical CD56^bright^ and CD56^dim^, further NK cell populations were identified. For instance, an anergic NK cell subset represented by CD56^−^ (CD56^−^/CD16^+^) phenotype pathologically expands during the course of HIV-1 infection [9]. Recently, an unconventional population of CD56^dim^/CD16^-^ (unCD56^dim^) NK cells was described, which had potent cytotoxic potential among blood-circulating NK cells, in patients affected by hematologic malignancies receiving haploidentical hematopoietic stem cell transplants (haplo-HSCT) [10,11].

The high cytotoxic activity of peripheral blood NK cells positively correlates with reduced cancer risk, thus indicating the critical role of NK cells in controlling the development of various types of tumor in vivo [12]. Numerous pre-clinical studies associated favorable NK cell survival with better clinical outcome and therapeutic response in different cancers [13,14,15]. However, the specific role of NK cells remains controversial and largely depends on distinct cancer types. Tumor-infiltrating NK cells are highly heterogeneous, even in the same type of cancer, and are characterized by an abundant expression of various activating and inhibitory surface receptors, due to the complexity of the tumor microhabitat [16]. Recently, CIBERSORT, an analytical analysis tool, divided NK cells into resting and activated cell subtypes, each contributing to the formation of the tumor microenvironment (TME) [17]. In tumors, alterations in antigen presentation in the context of specific alleles of HLA class I (i.e., HLA-C, HLA-E, or HLA-G), recognized by iNKRs, might lead to the impairment of the NK cell anti-tumor response [18]. Moreover, tumor-transformed cells can create an immune-suppressant microenvironment that is able to down-modulate the repertoire of aNKRs and further impair both NK cell effector functions and trafficking to tumor sites, as previously reviewed [14].

Thus, a better understanding of the mechanisms that shape NK cell phenotype and influence their antitumor activity in various types of tumors might provide therapeutic benefits.

### 2.2. DCs in Cancer Immunity

DCs are professional antigen presenting cells (APCs) with the unique ability to prime adaptive immune responses. As a result, DCs are essential for the activation of anti-tumor immune responses. Originating from bone marrow, DCs migrate to peripheral tissues through the blood. In the peripheral tissues, they sample antigens and migrate to lymph nodes, where they present the captured antigens to T cells. Depending on the signals sensed by DCs in peripheral tissues, antigen presentation is followed by either the activation of different arms of adaptive immunity or by the induction of T cell tolerance. This different behavior of DCs in shaping the adaptive immune response is also dependent on the different DC subset to which the DCs belong [19]. DCs are heterogeneous and can be divided into two major populations—conventional DCs (cDCs) and plasmacytoid DCs (pDCs). cDCs are myeloid cells that are further divided into two subsets—cDC1s and cDC2s. cDC1s develop under the control of the transcription factors (TFs) IRF8, ID2, and BATF3 [20], and selectively express the C-type lectin receptor CLEC9A, the cell adhesion molecule 1 (CADM1), CD141, and the chemokine receptor XCR1 [21,22]. cDC1s have an excellent cross-presentation ability that makes them highly efficient in priming cytotoxic T cells [23]. cDC1s play a crucial role in the activation of anti-tumor immune responses, also acting locally in the TME, where, by producing the chemokines CXCL9 and CXCL10, they attract tumor-infiltrating T cells in the cDC1-rich areas and sustain local T cell restimulation [24]. Due to these properties, cDC1s are crucial for the activation of effective anti-tumor immune responses, and their presence in the TME is associated with better prognosis across human tumors [25,26]. cDC2s develop under the control of the TFs IRF4, ID2, ZEB2, and KLF4 [19,20], and are characterized by a preferential expression of different markers, including CD1c, the α subunit of the high-affinity IgE receptor (FCER1A), CLEC10A, CD301, and the signal regulatory protein α CD172a [27,28,29]. cDC2s are specialized in the activation of naive T helper (Th) cells through the presentation of antigens in the context of HLA class II molecules [30]. They are more heterogeneous and more abundant in the blood and tissues than cDC1s. cDC2s express a wide variety of pattern recognition receptors on their surface, which enable these cells to sense a broad spectrum of different signals in peripheral tissues. In response to these signals, cDC2s produce different arrays of cytokines, which in turn polarize different Th responses [31]. In the context of anti-tumor immune responses, cDC2s play an important role as potent stimulators of naive Th cells, which are needed for the activation of optimal anti-tumor cytotoxic T lymphocyte (CTL) functions. It was demonstrated that the cooperation of CD4^+^ and CD8^+^ T cells enhances high-avidity tumor, antigen-specific CTLs, and improves the clinical response to cancer immunotherapy [32,33,34,35]. However, non-helped CTLs are impaired in several functions, including their ability to infiltrate the TME [36]. pDCs develop under the control of the TFs IRF4, IRF8, E2-2, and ZEB2 [19,20], lack myeloid markers and are characterized by the expression of CD303, CD304, and the IL-3R-α receptor CD123 [21]. Immature pDCs are mainly tolerogenic, which explain the observation that their presence in the TME is mainly associated with a poor prognosis across many types of human cancer [37,38,39,40]. Upon activation, pDCs excel in their ability to produce huge amounts of type I IFN [41]; they can present antigens and activate T cells, although this issue was long debated upon [42,43].

Despite the above-described functional specialization of the DC subsets, the efficiency of DCs to activate immune responses also relies on the interactions of each subset with other DCs. In the context of anti-tumor immune responses, accumulating evidences suggest that though needed for the activation of anti-tumor immune responses, cDC1s are not sufficient for optimal responses, and crosstalk between DC subsets is emerging as crucial for maximizing the activation of protective responses. In this context, if properly activated, pDCs in the TME provide an important contribution to the activation of effective anti-tumor immune responses, by producing high amounts of type I IFN, which in turn, activates cDCs by up-regulating their surface expression of costimulatory molecules. The close proximity of pDCs and cDC1s also suggests that the synergistic effects between these DC subsets might rely on cell-contact-dependent mechanisms [44,45]. Notably, the ability of pDCs to transfer antigens to cDCs through exosomes was recently demonstrated, indicating that pDCs can cross-prime naïve cytotoxic T cells through the involvement of cDC1s [46]. cDC1s can also collaborate with cDC2s for optimal CTL induction. In particular, Th1 cells activated by cDC2s can promote the activation of cDC1s by sustaining their upregulation of costimulatory molecules and production of IL-12 and IL-15, which in turn, are required for optimal CTL activation [46,47]. Moreover, cDC1s–cDC2s crosstalk might also sustain optimal Th1 activation, as demonstrated in infections and autoimmunity, where cDC2-induced Th1 responses are potentiated by IL-12 produced by cDC1s (reviewed in [47]). Notably, this important crosstalk between different DC subsets occurs not only in the tumor-draining lymph nodes, but also in the TME where the development of tertiary lymphoid structures (TLS) facilitates proper immune cell interactions [48,49]. As reported above, DCs also play an important role at the tumor site where, through the secretion of CXCL9 and CXCL10, cDC1s contribute to the recruitment and restimulation of T cells [24,26]. Because IFNs are required to stimulate the production of these chemokines by cDC1s, tumor-infiltrating T cells that secrete IFN-γ, namely cDC1-induced CTLs and cDC2-induced Th1 cells, establish a positive feedback loop that amplifies the cDC1-dependent recruitment of additional T cells [26]. Notably, as reported in detail below, these same cDC1-derived chemokines and cytokines are also involved in sustaining the crosstalk between DCs and NK cells.

Similar to other immune cells, DCs undergo profound changes during the complex interaction that occur between tumor cells and the immune system, in the multistep process of cancer immunoediting [50]. During the elimination phase, immunostimulatory DCs activate robust tumor-specific cytotoxic immune responses that destroy tumor cells. During the equilibrium phase, immunostimulatory DCs contribute to keep resistant tumor cells in a state of dormancy, thus, preventing tumor cell outgrowth [51]. During the escape phase, tumors acquire new properties that elude immune recognition and destruction and promote the development of an immunosuppressive TME [52]. Tolerogenic DCs contribute to the escape phase by promoting tumor-specific immune tolerance, and participating in the development and maintenance of an immunosuppressive TME [53,54]. Notably, numerical and functional impairments of DCs in patients with different types of cancer were reported not only in the TME, but also in DCs circulating in the peripheral blood [50,55,56,57], indicating that tumors can affect DCs far beyond the local environment.

### 2.3. DC-Mediated Control of NK Cell Activation

Since the first evidence of NK–DC interactions reported by Fernandez et al., showing the ability of DCs to trigger the NK cell antitumor response, other studies investigated the interactions between these two types of cells [58,59]. DCs were shown to be required for the activation of NK cells in response to viral infections, such as cytomegalovirus (CMV) [60,61,62] and herpes simplex virus type 1 (HSV-1) [63,64,65]. Moreover, multiple studies reported the relevant role of DCs in the activation of NK-cell-mediated antitumor activity [66,67]. The mechanisms through which DCs activate NK cells involve a combination of secretion of inflammatory cytokines and direct cell-to-cell contact that promote NK cell proliferation, cytokine production, and induction of cytolytic activity. NK proliferation and survival were shown to be mediated by DCs through the release of IL-15 [8]. IL-15 *trans*-presentation by DCs is required for NK cell priming [68,69,70], and IL-15 *cis*-presentation by NK cells contribution to NK cell activation through the production of IFN-β by DCs [71]. A strong activator of NK effector functions is IL-12; DCs-derived IL-12 stimulates NK cells to produce IFN-γ [2,8]. A similar role is exerted by IL-18, which synergizes with IL-12 to induce IFN-γ secretion by NK cells [64]. IL-18, alone or in combination with IL-12, enhances NK cell cytotoxicity in both microbial infection and the antitumor response [59,67]. In addition, type I IFNs (IFN-α/β), secreted by DCs play a crucial role in the induction of NK cell cytotoxicity [72,73]. In this context, pDCs, the major producers of type I IFNs in the blood, are the main subset affecting NK cell cytolytic activity [72,74]. Thus, cytokines secreted by DCs are important players in the DCs–NK cells interactions; however, direct cell-to-cell contacts are required for optimal NK cell activation by DCs. The establishment of an immunological synapse is essential for the complete activation of NK cells [67,69]. MHC class-I-related (MIC) A and B expressed on DCs upon IFN-α stimulation induce NK cell activation through NKG2D recognition [75]. Moreover, viral-infected DCs engage both NKG2D and NKp46 receptors on NK cells to trigger their effector functions [76]. pDCs activated by CpG-oligodeoxynucleotides (CpG-ODN) express the ligand for the glucocorticoid-induced tumor necrosis factor receptor (GITRL), whose receptor is expressed by activated NK cells, thus promoting NK cell cytotoxicity [77]. Cytoskeleton remodeling and lipid raft mobilization are also involved in the formation of DCs–NK cells stimulatory synapses, as they promote the polarized secretion of IL-12 by DCs toward NK cells [78].

Most findings related to the effect of DCs on NK cell activation are based on monocyte-derived DCs, generated in vitro through cytokine stimulation, and little is known about the interaction between human DCs and NK cells in vivo. Notably, both soluble factors and direct cell-to-cell contact between blood CD83^+^ DCs and NK cells enhance the NK cell cytolytic activity and the tumoricidal potential [79,80]. Moreover, lipopolysaccharide (LPS)-activated 6-sulfo LacNAc^+^ (slan) DCs are able to activate NK cells by inducing CD69 expression, IFN-γ secretion, and tumor-directed cytotoxicity [81], thus confirming a regular circuit between human DCs and NK cells occurring in vivo, in which the resting NK cells require DCs for activation.

### 2.4. NK-Cell-Mediated Control of DC Activation

The DC–NK crosstalk is a bidirectional interaction since NK cells also affect DC maturation and effector functions. To limit the supply of DCs at sites of inflammation and to control adaptive immune responses, NK cells kill autologous immature DCs (iDCs) through direct DCs–NK interactions [82]. The susceptibility of iDCs to NK cell lysis relies almost entirely on the NKp30 activating receptor, and downmodulation of NKp30 upon tumor growth factor (TGF)-β stimulation inhibits the NK-cell-mediated killing of DCs. [83,84]. However, only a small subset of NK cells showed the ability to kill DCs; this cell subset is characterized by the expression of the inhibitory receptor NKG2A and the absence of KIRs [85]. Accordingly, iDCs displayed downregulation of the surface HLA-E, which explains their susceptibility. In addition to NKp30, DNAM-1 and CD54 are involved in the NK-cell-mediated killing of DCs [86,87]. Recently, it was shown that the engagement of NKp30 and DNAM-1 expressed on NK cells can not only lyse iDCs but can also induce the Th2-polarizing properties of DCs [88].

NK cells have an important function in the process of DCs maturation. Although at high NK/DCs ratios, the interaction between NK cells and DCs results in DCs killing, at low ratios, NK cells can stimulate DCs maturation [89]. NK cells regulate the maturation and cytokine release of different types of DCs including myeloid DCs, pDCs, and slanDCs [74,81]. In this context, both cytokines, TNF-α and IFN-γ, and cell-to-cell contact are important for NK-cell-mediated DC activation [74,89]. In addition, a critical role is displayed by NKp30, whose engagement upon NK/DCs interaction induces cytokine-dependent DCs maturation [90].

Several lines of evidence suggest that NK cells can have a helper function and support DCs to drive T cell differentiation. In particular, NK cells can induce stable type-1-polarized DCs (DC1), which allow the development of Th1- and CTL-dominated type 1 immune responses [91]. The ability of NK cells to promote the Th1 polarization of DCs depends on cytokines release. In particular, IL-12- or IL-2-activated NK cells induce the maturation of DCs, which prime Th1 cells, whereas NK cells exposed to IL-4 impaired efficient DCs Th1 priming and might favor tolerogenic or Th-2 responses [92,93]. Recently, it was shown that NK cells can also prime inflammatory DCs to drive the differentiation of type 17 CD8^+^ T cells (Tc17), which have the capacity to produce IFN-γ and IL-17 [94].

**Figure 1:**
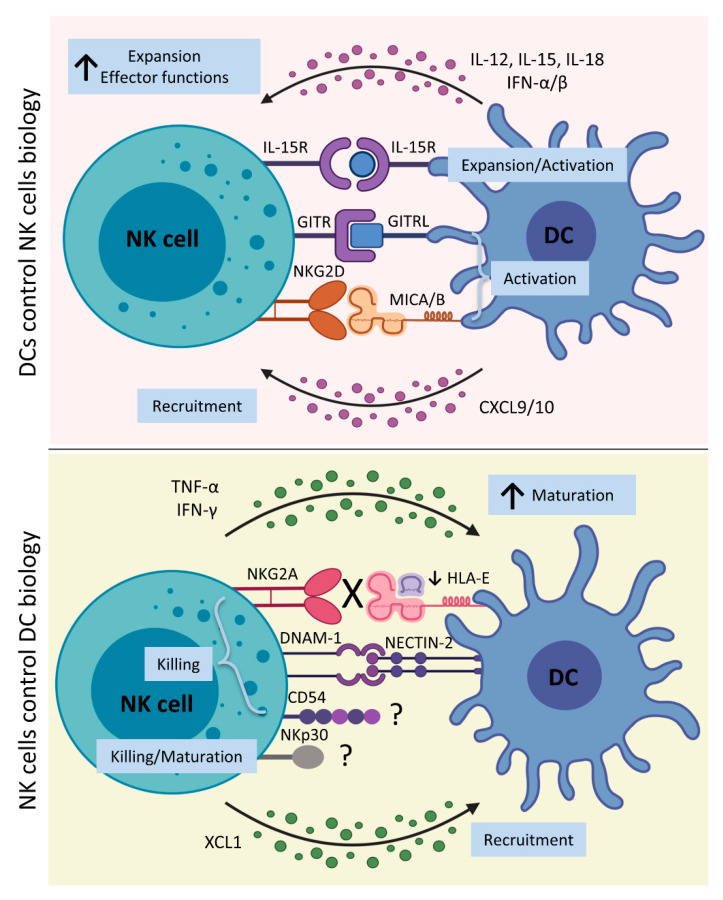
DC–NK cell interplay. **Upper panel**: After exposure to several stimuli recognized by a wide variety of pattern recognition receptors expressed by DCs, they release different cytokines, like IL-12, IL-15, IL-18, and IFN-γ. IL-15 *trans*-presentation by DCs is required for NK cell priming, and IL-15 *cis*-presentation by NK cells contributes to NK cell activation through the production of IFN-β by DCs. IL-12 and IL-18 stimulate NK cells to produce IFN-γ and enhance NK cell cytotoxicity. Moreover, direct NK–DC contact is required for optimal NK cell activation by DCs. The interaction of NKG2D with MICA/B expressed by DCs upon IFN-α simulation, and the interaction of GITR with its ligand on DCs also lead to NK cell activation. Moreover, DCs release chemokines, such as CXCL9/10, which further recruit NK cells at the site of inflammation. **Lower panel**: Activated NK cells produce IFN-γ and TNF-α, which promote maturation of DCs that are able to drive Th1- and cytotoxic T cell development. Additionally, the NKp30 engagement is involved in this process. To increase DC recruitment at the site of inflammation, NK cells release XCL1, which binds to XCR1 expressed by DCs. To limit the supply of DCs at sites of inflammation and control excessive adaptive immune response, NK cells that express the inhibitory receptor NKG2A and lack activating KIRs can kill autologous iDCs that display a reduction in HLA-E expression. In particular, the direct NK–DC interaction, through NKp30, DNAM-1, and CD54, on NK cell surface, and their ligands on DC surface, induces the killing of iDCs. Notably, NKp30 and CD54 ligands on DCs surface are still unknown, as indicated in the figure by a question mark. [CXCL10, Chemokine (C-X-C motif) ligand 10; CXCL9, Chemokine (C-X-C motif) ligand 9; DCs, dendritic cells; DNAM-1, DNAX Accessory Molecule-1 or CD226; GITR, glucocorticoid-induced tumor necrosis factor receptor; GITRL, glucocorticoid-induced tumor necrosis factor receptor ligand; HLA-E, Major Histocompatibility Complex, Class I, E; iDCs, immature dendritic cells; IFN-α, interferon α; IFN-β, interferon β; IFN-γ, interferon γ; IL-12, interleukin 12; IL-15, interleukin 15; IL-15R, interleukin 15 receptor; IL-18, interleukin 18; KIRs, Killer-cell immunoglobulin-like receptors; MICA/B, MHC class-I-related protein A/B; NECTIN-2, Nectin Cell Adhesion Molecule 2 or CD112; NK cells, natural killer cells; TNF-α, tumor necrosis factor α; XCL1, Chemokine (C motif) ligand 1; and XCR1, X-C Motif Chemokine Receptor 1]. Created with BioRender.com (accessed on 16 March 2021).

Recent studies clearly elucidated the essential role of the crosstalk between NK cells and DCs occurring during immune responses, summarized in Figure 1. This interaction results in the development of an efficient innate response and a potent adaptive immune response through bilateral NK–DC maturation and activation.

## 3. Specialized NK Cell and DC Subsets in The Liver

### 3.1. Biological Complexity of Hepatic NK Cells

Other than circulation, NK cells are found in different organs. In humans, CD56^dim^ cells predominate in bone morrow, spleen, and subcutaneous adipose tissue; conversely, the CD56^bright^ cell subset is found in much higher proportions in tissues such as intestinal mucosa, kidney, uterus, and liver [95,96]. Thus, the high frequencies of NK cells in several peripheral tissues extend traditional NK cell dichotomy to a much broader spectrum of NK cell diversity related to the tissue-specific imprinting in physiological and pathological conditions. 

Liver (lr)-NK cells represent the most abundant population among all intrahepatic lymphocytes, which can reach up to 50% [97,98]. For the first time, lr-NK cells were described in mouse. However, this concept was rapidly confirmed in human liver, where NK cells are represented by heterogenous phenotypic profiles with either cytotoxic or tolerogenic effector functions. The CD56^bright^ cell subset is highly enriched in the liver parenchyma [99,100,101]. The specific phenotype of human lr-CD56^bright^ cells is characterized by the constitutive expression of the chemokine receptors CXCR6 and CCR5, and the tissue residence marker CD69 [99,102,103]. The constitutive expression of CXCR6 and CCR5 on lr-NK cells appears to be important in the mechanisms of recruitment and retention of NK cells in the liver. The engagement of these chemokine receptors following binding with their cognate ligands (i.e., CXCL16 and CCL3-CCL5, respectively), expressed by cholangiocytes, sinusoidal endothelial cells, hepatocytes, and Kupffer cells (KCs) (i.e., liver-resident macrophages), is associated with liver homing [97,99]. However, another mechanism restricted to hepatic trafficking of NK cells was proposed, involving VAP-1 expression on sinusoidal endothelial cells, which in turn, binds the Siglec-9 molecule expressed on the blood-circulating NK cells [104,105]. Lr-NK cells revealed the specific transcriptional profile characterized by a higher expression level of transcription factors EOMES and HOBIT, and in contrast to peripheral blood NK cells, a low level of T-bet [102,106,107]. 

Liver is the organ in which for the first time NK cells were identified with the memory-like features of adaptive immunity, which include clonal-like expansion and generation of long-lived memory populations capable of enhanced recall responses [108]. Human memory-like NK cells were found in HCMV infection, which induces the expansion of the specific NKG2C^+^ NK cells that are able to produce a higher amount of IFN-γ after being re-challenged with the same virus [109,110,111]. These expanded NKG2C^+^ NK cells have a unique surface phenotype, with preferential expression of the maturation marker CD57 and inhibitory KIRs, and reduced expression of the NCRs and the transcription factor PLZF [109,111,112,113].

The complexity of human lr-NK cell subsets was recently confirmed using unbiased analysis at the single cell level with both multiparametric flow cytometry and RNA sequencing (RNA-seq) technology [114,115,116]. These studies demonstrated the CXCR6^+^ phenotype of lr-NK cells expressing EOMES, along with tissue residency markers CD69, CD49a, and CD103, and several inhibitory and activating molecules such as NKG2A, KLRB1, GZMK, and CD160. In addition, CXCR6^-^ CD103^-^ CD69^low^ was described with the memory-like NKG2C^+^CD38^low^KIR^+^PLZF^−^ phenotype.

The hepatic immune system must simultaneously respond to harmful and remain tolerant to many antigens derived from the gut [117,118,119]. Immune tolerance in the liver is mediated by a number of specialized APCs. Among them, DCs and KCs are critical in the maintenance of NK cell tolerance in the liver, mainly by producing IL-10 and TGF-β [120,121,122]. Moreover, increase in tolerogenic NK cells could be prompted by apoptotic cells that are able to expand the NK TGF-β-producing ability to suppress their autocrine IFN-γ production [123].

Importantly, the tolerance of lr-NK cells is simultaneously maintained with their high effector potential. Lr-NK cells present high levels of granzymes, perforin, and IFN-γ [124,125]. In addition, the high expression levels of TRAIL and FasL molecules suggest preferential direct cellular-dependent killing of the target cells [126]. Moreover, lr-NK cells, as compared to the peripheral blood NK cells, are more efficient producers of TNF-α, GM-CSF, and IL-2, which are key players in inflammatory responses [126,127,128]. This effector potential of hepatic NK cells is largely documented in acute hepatitis B virus (HBV) and hepatitis C virus (HCV) infections. The early NK cell activation during viral infection, mediated by IFN-α/β and cytokines such as IL-12, IL-15, and IL-18, contributes to both the initial control of infection and to the development of an efficient adaptive immune response [97,129]. However, viral clearance correlates with increased frequency and activation of peripheral blood NK cells, which result in a higher cytotoxicity and secretion of IFN-γ [130,131].

Hepatic NK cells play an important role in liver regeneration after tissue damage. Hepatocyte proliferation significantly decreased in mice models lacking NK cells [132]. During fibrosis, resolving hepatic NK cells directly kill activated stellate cells (SCs), inducing SCs apoptosis in NKG2D-dependent and TNF-α-related manners [133]. Interactions of NK cells with different liver-resident cells (i.e., KCs, fibroblast, and stem cells) produce the secretion of different cytokines and chemokines, including CXCL7, CXCL2, CCL5, and IL-8, which are able to induce the proliferation and regeneration of hepatic tissue, although their excessive activation can inhibit rather than promote liver regeneration [134,135].

Despite the liver being considered a preferential tissue for NK cells residency, several questions still remain unanswered. In particular, given the high heterogeneity of lr-NK cells, further studies are needed to investigate their specific role in both homeostatic and pathological conditions.

### 3.2. Heterogenicity of Liver DCs

In the healthy liver, constantly changing metabolic and tissue remodeling activity, combined with regular exposure to microbial products, result in persistent and regulated inflammation and immune responses, the failure of which leads to chronic infection, autoimmunity, and tumor growth [1]. The hepatic blood system allows the rapid exchange of material from the blood rich in pathogen-associated molecular patterns (PAMPs) and damage-associated molecular patterns (DAMPs) into hepatocytes, which sense these molecules through the expression of pattern recognition receptors (PRRs). Upon binding, this material is phagocytosed and subsequently degraded by hepatocytes and KCs, without mounting the inflammatory response that usually accompanies PRR signaling. An important role in the maintenance of hepatic tolerance is exerted by resident myeloid cells, KCs, and DCs, by producing anti-inflammatory cytokines such as IL-10, and downregulating co-stimulatory molecules, limiting the adaptive immune response [136]. These resident immune cells populate the liver sinusoids and the sub-endothelial compartment, the space of Disse, where lymph collects and flows into lymphatic vessels running along the portal tract. In particular, during the steady state, DCs are localized around portal triads. In response to infection, DCs migrate from the parenchyma via the space of Disse to the portal area where they interact with T cells [137].

Whereas several liver-resident immune cell populations, like KCs, are well recognized, the full spectrum of immune cells resident within the liver, like DCs, is still unclear, in particular in humans, where further studies are needed to define phenotypically distinct liver-resident immune cell populations. From the first immunohistochemistry studies on healthy liver resections to more recent flow-cytometric applications, several authors documented the presence of lineage DCs, including cDC1s, cDC2, and pDCs. Concerning the molecular profile and functions of DC subsets in the human liver, information is lacking, but what emerged by combining data from murine and human DCs is that cDCs and pDCs share a tolerogenic profile [136,138,139]. From murine data, it was observed that cDCs are able to produce prostaglandin E2 (PGE2) and IL-10, and that the liver microenvironment is crucial for programming hematopoietic progenitor cells to develop into tolerogenic DCs in situ, a process that might contribute to the maintenance of hepatic tolerance through the inhibition of T cell proliferation and the induction of T_regs_ [140,141]. In humans, in the healthy hepatic resections from benign disease, primary cancer, and metastatic disease, a significant increase of cDC2s was observed, as compared to blood. Hepatic cDCs were also characterized by a lower expression of costimulatory molecules as compared to the spleen, and produced greater amounts of IL-10 when stimulated with LPS as compared to blood DCs. In mixed lymphocyte reaction (MLR) experiments, researchers observed that bulk cDCs induced a lower proliferation of allogenic T cells, promoting their hyporesponsiveness [140]. However, a partially different situation was observed when analyzing DC subsets in perfusates from livers of healthy donors. Kelly et al. observed an inverted scenario compared to that observed by Bamboat et al.—they found an increase in cDC1s and a decrease in cDC2s in liver perfusates as compared to blood. From a functional point of view, DCs in liver perfusates seemed to be less tolerogenic as compared to those of the parenchyma. Indeed, it was observed that cDC1s produce less IL-10; more IL-1β, CXCL10, TNF-α, IL-6, and IL-8; and together with cDC2s, were able to induce allogenic T cells proliferation [142]. Interesting evidence of the tolerogenicity of DCs in the liver was provided by Ibrahim et al., who observed both in mice and humans the presence of two subsets of liver DCs on the basis of lipid content (low-DC and high-DC). These are unique in the liver (none in the spleen, kidney, LNs, or skin), and in particular, high-DCs were found to be more immunogenic, being characterized by high expression levels of co-stimulatory molecules (CD40, CD80, and CD86) and CD1d, as compared to low-DC, which conversely, were observed to be more tolerogenic [143].

## 4. NK–DC Crosstalk in Primary Liver Cancer

### 4.1. NK Cells in Liver Cancer

As an immune-tolerant organ, the liver is predisposed to tumorigenesis. Primary liver cancers, HCC and ICC represent the sixth most commonly diagnosed cancers. Several studies reported the involvement of NK cells in the immune surveillance of primary and metastatic liver malignancies [144,145]. A higher number of lr-NK cells predicts better outcome in HCC patients in the early stage of the disease in terms of tumor growth and better patient survival [146,147,148]. In particular, expression of NKG2D ligands might boost NK-mediated antitumor activity in HCC patients [149,150]. However, NK cells appear to be poorly efficient in controlling advanced HCC, due to their lower frequency and aberrant effector functions found in both blood and lr-NK cells. A lower occurrence of CD56^dim^ and CD56^bright^ NK cells was observed in tumor-infiltrated areas as compared to healthy tissue [148,151,152]. In part, the loss of NK cells in HCC was associated with an increased frequency of T_reg_ and a higher concentration of immunosuppressive IL-10 cytokine [148]. In addition, lr-NK cells exhibit poor capacity to produce cytokines (IFN-γ and TNF-α) and kill tumor targets. Several phenotypic features and multiple mechanisms were proposed to explain NK cells’ impairment in HCC. Among them, increased expression of several immune checkpoint molecules, including programmed cell death protein (PD-1) and NKG2A, was found on lr-NK cells [153,154]. PD-L1 and MHC class I molecules were found to be highly present on HCC cells [155,156]. Lr-NK cells in HCC patients with advanced-stage disease showed a prevalent expression of the specific NKp30 splice variant, resulting in inhibitory NKp30-mediated functionality [157]. The direct contact with tumor-associated macrophages might induce rapid NK cell exhaustion through CD48/2B4 or NKp30 interaction [152,158]. In addition, several immunomodulators, such as TGF-α, PGE2, or indoleamine 2,3-dioxygenase (IDO), released in the tumor microenvironment and contributing to HCC progression show an immunosuppressive impact on NK cells [159]. Thus, the potential of NK cells to control HCC development is largely reduced by different strategies exerted by HCC, to evade the NK-cell-mediated immune surveillance.

Among the main predisposing risk factors of HCC, there are chronic HBV and HCV infections [160]. In the acute phase of HCV and HBV infection, the NK cells display increased IFN-γ production and increased cytotoxicity, although, the specific contribution of NK cells to antiviral response is still unknown [161,162]. During this period, HCV patients are typically clinically asymptomatic and, without treatment, two-thirds of the infected patients develop chronic disease. Similarly, HBV infection is characterized by a lack of obvious symptoms and liver damage that even lasts for several years. With the development of infection, the clearance of viral infection correlates with elevated levels of IFN-γ and TNF-α in the liver. Persistent HBV and HCV infections have a remarkable impact on the hepatic NK cell repertoire, profoundly affecting their effector functions and resulting in preserved NK cell cytotoxicity and impaired IFN-γ production [163,164]. Based on a model proposed by Miyagi et al., NK cells produce IFN-γ in the early phase of a virus infection due to their constitutively high STAT4 expression [165]. Chronic exposure to type I IFN results in increased STAT1-dependent cytotoxicity and decreased STAT4-dependent IFN-γ production. The altered functional phenotype of NK cells in chronic HCV and HBV infection might therefore facilitate chronic inflammation via killing of infected cells but not allowing viral clearance due to impaired IFN-γ production. Of note, the preserved cytotoxic potential of lr-NK cells in chronic HBV infection contributes to the killing of autologous virus-specific CD8^+^ T cells, thus impairing adaptive antiviral immunity [166]. These alterations in NK cells, such as the persistent deregulation of IFN-γ signaling, are associated with persistent HCV and HBV infection, liver injury, liver fibrosis, and liver carcinogenesis and thus, presumably contribute to liver disease progression [167].

Numerous studies assessed the protective role of NK cells in ICC development. The use of in vitro cytokine-activated NK cells in combination with cetuximab, the monoclonal antibody (mAb) against epidermal growth factor receptor (EGFR), showed benefits, resulting in a higher ADCC response against human ICC cells [168]. In addition, several infusions of in vitro expanded human NK cells into ICC xenograft tumor-bearing mice showed high NK-cell-mediated cytolytic response with inhibition of tumor growth [169]. Recently, the elevated intra-tumoral expression of CXCL9, an IFN-γ inducible chemokine, was associated with a higher number of tumor-infiltrating NK cells, leading to favorable postoperative survival in patients with ICC [170]. Additionally, elevated expression of the NKG2D ligand correlates with improved overall ICC patient survival [171]. Although these findings hold promise, similar to HCC, strategies with the aim of evading NK cell immune surveillance in ICC were also reported. For instance, ICC cells induce apoptosis of NK cells via the Fas/FasL cell death pathway, and escape the inflammatory response by upregulating the antiapoptotic c-FLIP system [172]. Several single nucleotide polymorphisms (SNPs) located within the NKG2D receptor gene (*KLRK1*) were linked to defective NK-cell-mediated anti-tumor activity and a higher risk of cancer [173].

The emerging role of NK cells in primary liver tumors will likely open new opportunities for therapeutic strategies to restore or carry out NK cell-impaired antitumor activity.

### 4.2. DCs in Liver Cancer 

Effective immune responses against tumors are based on the activity of DCs that can recognize, process, and present tumor antigens. DCs are considered a promising tool for novel immunotherapeutic agents; however, dysfunctions in DCs activity were described in various tumors, including primary liver cancers.

In HCC patients, lower numbers of DCs expressing CD83, which is considered a maturation marker, were observed in tumor lesions, as compared to healthy controls. These cells localized in non-cancerous cirrhotic nodules and were absent in cancer nodules [174].

In another study, the presence of pDCs and DC-LAMP^+^ was assessed in lymph nodes of HCC patients. HCC patients with chronic viral hepatitis showed higher numbers of pDCs and lower numbers of cDCs in lymph nodes, as compared to healthy controls. However, the increased numbers of pDCs in lymph nodes was not accompanied by an increased number of cells producing IFN-α. In these conditions, pDCs preferentially stimulate Th2 or T regulatory differentiation, therefore suppressing the priming of tumor-specific T cells in hepatic lymph nodes [175]. Increased numbers of pDCs were also observed in the core lesion of HCC patients, as compared to the tumor-free areas of the liver. Tumor-infiltrating pDCs were found to co-localize with type 1 regulatory T (Tr1) cells and were the main population expressing ICOS-L. The interaction of ICOS-L with ICOS, expressed on Tr1, could induce the activation of Tr1 and their production of IL-10 [176]. All these observations point to a central role of pDCs in driving tumoral immunosuppression. 

In the peripheral blood of HCC patients, several studies observed a reduction in the percentage of circulating pDCs and cDCs, as compared to healthy controls [177,178,179]. In particular, Beckebaum et al. [178] observed that HCC patients were characterized by a reduction in circulating pDCs and both cDC subsets, cDC1s and cDC2s; moreover, all circulating DC subsets exhibited lower expression of HLA-DR and costimulatory molecules CD80 and CD86. Compared to healthy subjects, HCC patients presented a higher concentration of serum IL-10, which inversely correlated with the number of circulating DC subsets and their expression of costimulatory molecules, suggesting that circulating DCs in HCC patients mainly consist of immature cells and that the increased systemic levels of IL-10 might directly account for the alterations in the frequency and maturity of DC subsets.

In a different study, however, Ritter et al. observed comparable levels of circulating pDCs and cDCs in HCC patients, as compared to healthy adults [180]. They hypothesized that these discrepancies could be based on the Child-Pugh score evaluation of HCC patients, which was not considered in the other studies mentioned above. This finding suggests that the Child-Pugh score could be a major factor in DC immunoregulation.

Monocyte-derived DCs obtained from HCC patients resulted in lower expressions of HLA-DR, CD80, and CD86; lower production of IL-12; and higher production of nitric oxide (NO), as compared to healthy controls. NO was shown to inhibit T cells proliferation ability and might be responsible, together with low IL-12 production, for low allo-stimulatory capacity in allogenic MLR assays [181,182]. Nonetheless, these studies were based on in vitro generated monocyte-derived DCs, which present significant transcriptional and functional differences, as compared to the naturally occurring peripheral blood DCs.

In a recent work, Zhang et al. [183] dissected the landscape of immune cells in HCC patients with single-cell RNA-seq. cDC1s and cDC2s were found to be enriched in HCC core lesions. Another cluster that did not correspond to any classical DC subsets in the blood, LAMP3^+^ DCs, was enriched in the HCC core lesions. It was characterized by the expression of maturation markers LAMP3, CD80, and CD83, and by the migration marker CCR7. The presence of LAMP3^+^ DCs was validated in other cancer types [184,185] and in HCC patients with flow cytometry analysis. Altogether, the authors suggested that LAMP3^+^ DCs could represent a common subset in tumors that might mature from cDCs and have the ability to migrate toward lymph nodes. However, further investigations are needed to elucidate the potential role of this subset in various tumor contexts.

To date, little is known concerning the role of DC subsets in ICC. Lower numbers of circulating cDCs and reduced frequencies of TNF-α producing pro-inflammatory cells were observed in ICC patients, as compared to healthy controls [179], promoting a role for the TME that can influence DCs activity both at the local and systemic level. Indeed, it was also observed in tumors from the gastrointestinal tract that the tumor-conditioned media could inhibit TNF-α secretion in moDCs [186].

In another study, CD83^+^ DCs were mainly found at the invasive front of the tumor. The number of CD83^+^ DCs was considered a marker of good prognosis, and their density positively correlated with the number of CD4^+^ or CD8^+^ T cells [187], thus, suggesting that higher frequencies of mature tumor-infiltrating DCs favor the activation of tumor-specific immunity.

### 4.3. NK–DC Crosstalk in Liver Cancer

The NK–DC crosstalk shapes both the innate and adaptive antitumor responses. However, in primary liver cancers, both populations show various effector dysfunctions, with the consequent impairment of their reciprocal interaction and tumor control, as summarized in Table 1.

The altered NK–DC interaction is critical for the establishment of chronic HBV and HCV infections, which are one of the main risk factors for the development of HCC. NK cells isolated from patients with chronic HCV were not able to activate DCs in contrast to NK cells derived from healthy individuals, which instead induced maturation and activation of DCs [188]. The involved mechanisms possibly rely on a higher expression of NKG2A and an increase in production of IL-10 and TGF-β by NK cells, when co-cultured with hepatic cells. Impaired IL-15 production, observed in HCV-infected patients, causes the aberrant expression of MICA/B on DCs and a subsequent lower NKG2D-mediated NK cell activation [189].

Activation of pDCs during viral infections regulates IFN-γ production by human NK cells [190,194]. However, HBV infection is characterized by pDCs with defective responses to stimulation through the Toll-like receptor 9 (TLR9) ligand with the consequent aberration of the NK cell response [190].

An increased level of the immunosuppressive cytokine IL-10 characterizes the serum of HCC patients, which correlates with a decrease in frequency and altered maturation status of DCs [178]. The reduced number of DCs could be due to their lysis by autologous NK cells, as DCs exposed to IL-10 were shown to be more susceptible to NK cell killing [195]. Conversely, HCC patients show increased concentrations of the soluble form of MICA (sMICA), highly expressed on malignant cells in chronic disease [191]. The elevation of sMICA was associated with downregulated NKG2D expression and impaired activation of NK cells. Consequently, maturation and activation of DCs were completely abolished when NK cells were pre-treated with sMICA-containing serum [192]. As already discussed, chronic exposure to HBV increases TGF-β secretion, which also reduces the expression of NKG2D on NK cells, thus further impeding their ability to activate DCs [196].

Another molecule involved in the impairment of NK activation is α-fetoprotein (AFP), a tumor-associated antigen in HCC. Yamamoto et al. showed that AFP inhibits the production of IL-12 by DCs, which subsequently reduces the NK cytotoxic activity against tumor cells [193]. Moreover, AFP affected DC maturation, thus suggesting that high levels of AFP on the tumor side might induce killing of iDCs by NK cells, as mentioned above [82].

Recently, further exploration of the NK–DC interaction in HCC was extended to single-cell analysis using RNA-seq technology. By assessing the ligand–receptor pairs, it was predicted that LAMP3^+^ DCs could interact with NK cells via IL-15 and NECTIN, encoded by the *NECTIN2* gene [183]. Interestingly, NECTIN expressed on LAMP3^+^ DCs might interact with DNAM-1 (CD226) expressed on the blood-circulating NK cells, conferring an activating signal. Conversely, LAMP3^+^ DCs expressing NECTIN might interact with TIGIT expressed on lr-NK cells, conferring an inhibitory signal. This suggests that LAMP3^+^ DCs might regulate distinct NK cell subsets toward opposite directions.

The knowledge of the NK–DC crosstalk in ICC patients is still limited; however, the decreased frequency of TNF-α-producing DCs suggests a possible impairment in the activation of NK cells [179]. However, more studies are needed to investigate the interactions between NK cells and DCs in this primary liver tumor.

**Figure 2:**
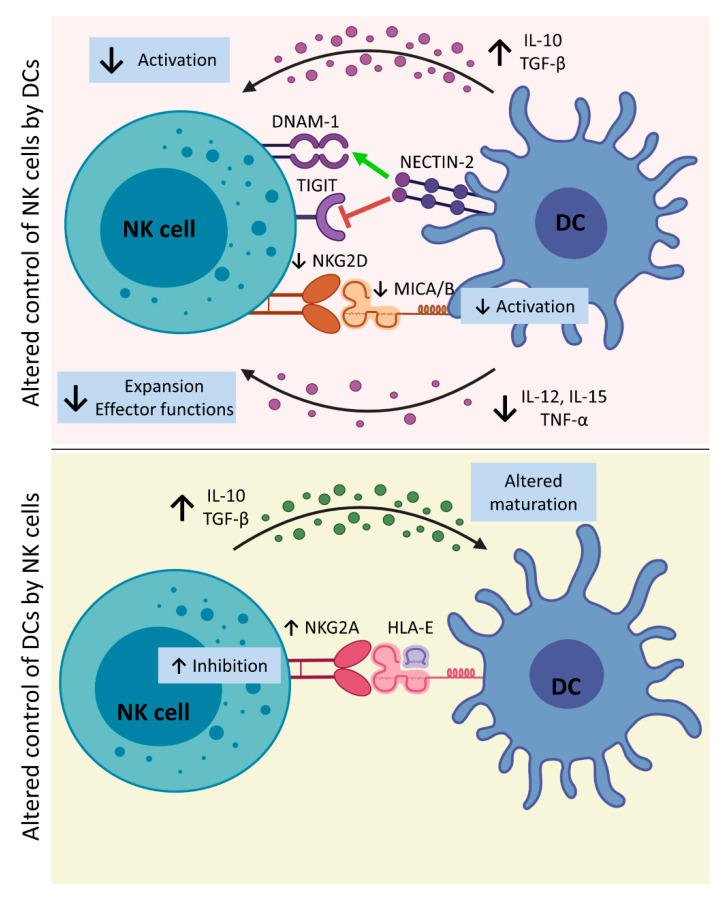
Altered DC–NK cell interplay in liver cancer. **Upper panel:** In the TME, DCs increase the secretion of IL-10 and TGF-β, which impair NK cell activation by the reduction of NKG2D expression on NK cells. Therefore, altered NKG2D–MICA/B interaction results in lower NK cell and DC activation. Moreover, the exposure to high levels of IL-10 and TGF-β induces a lower release of IL-12, IL-15, and TNF-α by DCs. In particular, impaired production of IL-15, causes the aberrant expression of MICA/B on DCs, and decreased expression of IL-12 by DCs induced by tumor AFP further harm NK cell activation. NK cell interaction by NECTIN-2 exposed on DCs can either block or increase NK cell activity through TIGIT or DNAM-1 interaction, respectively. **Lower panel:** Tumor-associated NK cells increase expressions of IL-10 and TGF-β that impair DC maturation, leading to the killing of iDCs by NK cells. Moreover, increased expressions of NKG2A on NK cells and HLA-E on DCs inhibit NK cell activity. [AFP, α-fetoprotein; DCs, dendritic cells; DNAM-1, DNAX Accessory Molecule-1 or CD226; HLA-E, Major Histocompatibility Complex, Class I, E; iDCs, immature dendritic cells; IL-10, interleukin 10; IL-12, interleukin 12; IL-15, interleukin 15; IL-18, interleukin 18; MICA/B, MHC class-I-related protein A/B; NK cells, natural killer cells; NECTIN-2, Nectin Cell Adhesion Molecule 2 or CD112; TGF-β, tumor growth factor β; TIGIT, T cell immunoreceptor with Ig and ITIM domains; TME, tumor microenvironment; and TNF-α, tumor necrosis factor α]. Created with BioRender.com (accessed on 16 March 2021).

Although a number of pathways and mechanisms exist for NK–DC interactions and reciprocal regulation during immune challenge, liver cancer induces profound dysregulation of both populations; summarized in Figure 2.

## 5. Implications of NK–DC Crosstalk in Liver Cancer Immunotherapy

**Figure 3:**
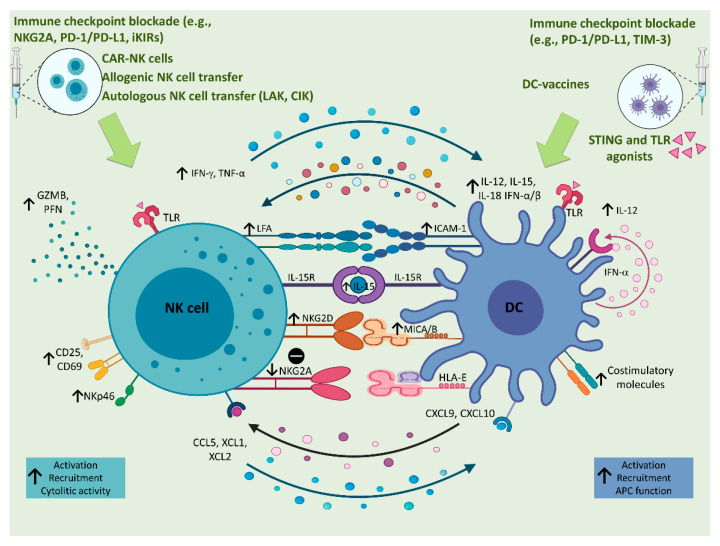
Implications of NK–DC crosstalk in liver cancer immunotherapy. Several immunotherapeutic approaches based on the administration of CAR-NK cells, allogenic or autologous NK cells or DC-vaccines are used in cancer patients to activate innate immune responses against tumor cells. The NK cell activation could also be reached through the administration of TLR agonists, like IQ and GDQ, which are, respectively, TLR7 and TLR7/8 agonists. NK cell activation on one hand leads to DC maturation, and on the other hand it is enhanced by the interaction with DCs, which in turn are activated by TLR agonists or STING. This bilateral activation involves the upregulation of CD69, CD25, NKp46, NKG2D, and the release of lytic enzymes (e.g., GZMB and PFN) by NK cells. On the other hand, activated DCs undergo upregulation of costimulatory molecules, NKG2D ligands (e.g., MICA/B) and an increased release of IFN-α, which in a positive loop, also contributes to the production of IL-12 that activates NK cells to produce high levels of IFN-γ and TNF-α. In addition, IL-15 and IL-18 sustain NK cell survival and activation that are also favored by the direct contact between NK cells and DCs through the interaction between NKG2D and its ligands, and LFA and ICAM-1, on NK cell and DC surface, respectively. Moreover, the reciprocal recruitment of both cellular populations is supported by the production of CXCL9 and CXCL10 by DCs and CCL5, XCL1, and XCL2 by NK cells. Other immunotherapeutic approaches, aimed at blocking inhibitory receptors, like the NKG2A, iKIRs, and the PD-1/PD-L1 axis, favor the activation of NK cells, consequently allowing DC maturation and activation, which could be also enhanced by the administration of immune checkpoint inhibitors against PD-1/PD-L1 or TIM-3. [CAR-NK cells, Chimeric Antigen Receptor natural killer cells; CCL5, C-C Motif Chemokine Ligand 5; CXCL10, Chemokine (C-X-C motif) ligand 10; CXCL9, Chemokine (C-X-C motif) ligand 9; DCs, dendritic cells; GDQ, Gardiquimod; GZMB, granzyme B; HLA-E, Major Histocompatibility Complex, Class I, E; IFN-α, interferon α; IFN-β, interferon β; IFN-γ, interferon γ; IL-12, interleukin 12; IL-15, interleukin 15; IL-15R, interleukin 15 receptor; IL-18, interleukin 18; iKIRs, inhibitory Killer-cell immunoglobulin-like receptors; MICA/B, MHC class-I-related protein A/B; IQ, Imiquimod; NK cells, natural killer cells; PD-1, programmed cell death protein 1; PD-L1, programmed death-ligand 1; PFN, perforin; STING, stimulator of interferon genes; TIM-3, T-cell immunoglobulin domain and mucin domain 3; TLR, toll-like receptor; TNF-α, tumor necrosis factor α; XCL1, Chemokine (C motif) ligand 1; XCL2, Chemokine (C motif) ligand 2; and XCR1, X-C Motif Chemokine Receptor 1]. Created with BioRender.com (accessed on 16 March 2021).

In the past decade, cancer immunotherapy produced an epochal change in the oncological treatment landscape. Several immunotherapeutic strategies were used to treat liver cancer, with most approaches focused on rescuing T cells from exhaustion, to unleash tumor-specific immune responses (reviewed in [197]). In particular, several clinical trials investigated the efficacy of mAbs directed against cytotoxic T lymphocyte antigen-4 (CTLA- 4), or against PD-1 or its ligand (PD-L1) in HCC patients. Despite encouraging responses observed in phase I/II studies, subsequent phase III trials failed to meet their primary survival endpoints [198]. Yet, the combination of anti-PD-L1 with antibodies directed against vascular endothelial growth factor (VEGF), which sustains tumor angiogenesis and mediates immunosuppression in the TME, succeeded in a phase III trial. Its efficacy and safety in different subgroups of HCC patients is currently under investigation (reviewed in [198]). The clinical data on immunotherapy in ICC are limited, and a number of clinical trials evaluating checkpoint inhibition combined with other therapeutic approaches are ongoing (reviewed in [199]). Notably, the safety of immune checkpoint blockade in ICC patients remains uncertain, as the risk of immune-mediated hepatobiliary toxicity might be high in these patients, due to their prevalent hepatic dysfunction and propensity for biliary obstruction. The compelling evidence of the critical role played by NK cells in liver cancer suggests that HCC and ICC might be an ideal target for NK-cell-based immunotherapies as well. Accordingly, as extensively reviewed elsewhere, recent studies and clinical trials used NK-cell-based strategies in liver cancer patients, including the administration of molecules that activate NK cell functions, the adoptive transfer of activated NK cells, and the use of mAbs that block the interaction between inhibitory receptors and their ligands [200,201]. As shown in Figure 3, under the effects of these therapeutic approaches, NK cells undergo upregulation of molecules involved in cytotoxic activity (including perforin, granzymes, and the cytotoxic receptor NKp46), increase the secretion of IFN-γ and TNF-α, upregulate the expression of the adhesion molecule LFA-1, and antagonize the expression of inhibitory molecules, including NKG2A. Interventions aimed at activating the NKG2D–MICA/B axis and delivering IL-15 to stimulate effector NK cells might represent other novel and interesting approaches [202]. All these NK-targeting strategies, used alone or in combination with other treatments, are expected to increase the number and the activation of NK cells at the tumor site.

DC-targeting immunotherapeutic strategies include DC vaccination, in vivo DC expansion, and in vivo DC reprogramming [203]. DC vaccination with exogenously expanded and activated autologous DCs was proven to have low toxicity but low efficacy in advanced HCC in different clinical trials, likely because the immunosuppressive TME circumvents the effects of immunotherapy [204,205,206]. Oncolytic immunotherapies, aimed at recruiting and activating DCs at the tumor site and triggering antitumor immune responses, provided disappointing results in HCC patients [204]. Increasing the number of tumor-infiltrating DCs by promoting DC expansion in vivo is an alternative approach for increasing DC function in antitumor immune responses. Indeed, the systemic administration of Flt3L was demonstrated to induce the systemic expansion of cDC1s in preclinical studies, and is currently under investigation in patients with breast cancer and lymphoma [203]. In vivo DC reprogramming could be achieved either by providing activatory signals or by blocking inhibitory signals. As DC subsets display differential TLR expression patterns [207], activation with TLR ligands allows the preferential activation of single DC subsets, although crosstalk between DC subsets is emerging to contribute significantly to antitumor immune responses [47]. Another activatory stimulus currently under investigation in preclinical models and clinical trials is represented by stimulator of interferon genes (STING) [203]. Inhibitory signals that can be targeted in order to reprogram in vivo DCs include VEGF, IL-10, and IDO. As DCs express immune checkpoints on their surface [55], immune checkpoint blockers targeting PD-1/PD-L1 and TIM-3 might also exert their efficacy in activating antitumor immune responses by in vivo DC reprogramming. The efficacy of these immunotherapeutic approaches in patients with liver cancer still needs to be investigated.

Although there is no direct evidence of the impact of NK- and DC-targeting immunotherapeutic approaches in NK–DC crosstalk in liver cancer, according to the above-described interactions occurring between NK cells and DCs in the liver, the effects of NK-targeting therapies on NK cells are expected to positively affect the recruitment and activation of DCs in the HCC and ICC TME. As recapitulated in Figure 3, activated NK cells can attract cDC1s through the production of CCL5, XCL1, and XCL2 [208]. Increased numbers of activated cDC1s in the TME can, in turn, sustain the recruitment and activation of other NK cells through the local release of IL-12, CXCL9, and CXCL10 [47,209]. On the other hand, the effects of DC-targeting immunotherapies on DCs are expected to positively affect the recruitment and activation of NK cells in the TME, through the production of IL-12, IL-18, and type I IFNs by DCs [59,67], through IL-15 *trans*-presentation [68,69,70], and through cell-to-cell contacts [67,69]. Indeed, a role for NK cells as helper cells in DC cancer vaccines was reported [210]. Relevant to liver cancer, a positive impact of DC vaccines on NK cells, characterized by increased expression of the activation markers CD25 and CD69 on circulating NK cells, was also reported in murine models of liver cancer [211,212,213] and in HCC patients [214]. Therefore, as summarized in Figure 3, there is increasing evidence that the activation of NK cells induced by NK-targeting strategies not only directly increases tumor cell killing by NK cells but also enhances antitumor adaptive immunity by promoting DC activation and the killing of immature DCs [215]. On the other hand, the activation of DCs induced by DC-targeting strategies not only directly increased the antigen-presenting function of DCs thus potentiating tumor-specific cytotoxic responses, but also potentiated the tumor killing function of NK cells [214]. In this scenario, combining the use of NK-targeting and DC-targeting approaches might represent a further strategy that might succeed to possibly overcome immunotherapy resistance in liver cancer patients.

## 6. Conclusions

Future studies would help increase our knowledge of the multiple interactions occurring between NK cells and DCs in liver cancer. The use of the highest-resolution methods for assessing the total cellular composition, the functional status, and the cellular localization of immune cells in the TME will allow the full characterization of NK–DC crosstalk, and the comprehension of its role in liver cancer development and the response to immunotherapeutic treatment.

## Figures and Tables

**Table 1:** Overview of findings on NK–DC crosstalk in liver cancer.

Pathology	Dysfunction	Effect	Reference
Chronic HCV infection	↑ NKG2A expression and ↑ production of IL-10 and TGF-β by NK cells	NK cells were not able to activate DCs	[188,189]
HCV infection	Aberrant expression of MICA/B on DCs due to impaired IL-15 production	↓ NKG2D-mediated NK cell activation	[188,189]
Chronic HBV infection	Defective responses of pDCs upon TLR9 stimulation	Aberrant NK cells activation	[190]
HCC	↑ sMICA is associated with ↓ NKG2D and impaired activation of NK cells	Abolished maturation and activation of DCs	[191,192]
HCC	AFP inhibits IL-12 production by DCs	↓ NK cytotoxic activity against tumor cells	[82,193]
HCC	LAMP3^+^ DCs expressing NECTIN might interact with DNAM-1 expressed on the blood-circulating NK cells; LAMP3^+^ DCs expressing NECTIN might interact with TIGIT expressed on lr-NK cells	Activating signal Inhibitory signal	[183]
ICC	↓ TNF-α-producing DCs	Impaired activation of NK cells	[179]

HCV = Hepatitis C virus, HBV = Hepatitis B virus, HCC = Hepatocellular carcinoma, and ICC = Intrahepatic cholangiocarcinoma. The ↑ symbol indicates an increase in frequency or expression levels and the ↓ symbol indicates a decrease in frequency or expression levels.

## Abbreviations

ADCC	Antibody-dependent cellular cytotoxicity
AFP	α-fetoprotein
APCs	Antigen presenting cells
CADM1	Cell adhesion molecule 1
cDCs	Conventional dendritic cells
CMV	Cytomegalovirus
CpG-ODN	CpG-oligodeoxynucleotide
CTL	Cytotoxic T lymphocyte
DAMPs	Damage-associated molecular patterns
DC	Dendritic cell
EGFR	Epidermal growth factor receptor
GITRL	Glucocorticoid-induced tumor necrosis factor receptor
Haplo-HSCT	Haploidentical hematopoietic stem cell transplant
HBV	Hepatitis B virus
HCC	Hepatocellular carcinoma
HCV	Hepatitis C virus
HLA	Human leukocyte antigen
HSV-1	Herpes simplex virus type 1
ICC	Intrahepatic cholangiocarcinoma
iDCs	Immature dendritic cells
IDO	Indoleamine 2,3-dioxygenase
IFN	Interferon
ILs	Interleukins
KCs	Kupffer cells
KIRs	Killer immunoglobulin-like receptors
LPS	Lipopolysaccharide
Lr-NK cells	Liver NK cells
mAb	Monoclonal antibody
MHC-I	Major histocompatibility complex class I
MICA/B	MHC-I-chain related protein A/B
MLR	Mixed lymphocytes reaction
NCRs	Natural cytotoxicity receptors
NK	Natural Killer
NKRs	NK receptors
NO	Nitric oxide
PAMPs	Pathogen-associated molecular patterns
PD-1	Programmed cell death protein
pDCs	Plasmacytoid dendritic cells
PGE2	Prostaglandin E2
PRRs	Pattern recognition receptors
SCs	Stellate cells
SLAN	6-sulfo LacNac
SNPs	Single nucleotide polimorphism
TFs	Transcription factors
TGF-β	Tumor growth factor beta
Th	T helper
TLR	Toll-like receptor
TLS	Tertiary lymphoid structures
TME	Tumor microenvironoment
TNF-α	Tumor necrosis factor alfa

## References

[1] Robinson M.W., Harmon C., O’Farrelly C. (2016). Liver Immunology and its role in inflammation and homeostasis. Cell Mol. Immunol..

[2] Vivier E., Raulet D.H., Moretta A., Caligiuri M.A., Zitvogel L., Lanier L.L., Yokoyama W.M., Ugolini S. (2011). Innate or adaptive immunity? The example of natural killer cells. Science.

[3] Chiossone L., Dumas P.Y., Vienne M., Vivier E. (2018). Natural killer cells and other innate lymphoid cells in cancer. Nat. Rev. Immunol..

[4] Karre K. (2008). Natural killer cell recognition of missing self. Nat. Immunol..

[5] Lanier L.L. (2008). Up on the tightrope: Natural killer cell activation and inhibition. Nat. Immunol..

[6] Di Vito C., Mikulak J., Mavilio D. (2019). On the Way to Become a Natural Killer Cell. Front. Immunol..

[7] Fehniger T.A., Cooper M.A., Nuovo G.J., Cella M., Facchetti F., Colonna M., Caligiuri M.A. (2003). CD56bright natural killer cells are present in human lymph nodes and are activated by T cell-derived IL-2: A potential new link between adaptive and innate immunity. Blood.

[8] Ferlazzo G., Pack M., Thomas D., Paludan C., Schmid D., Strowig T., Bougras G., Muller W.A., Moretta L., Munz C. (2004). Distinct roles of IL-12 and IL-15 in human natural killer cell activation by dendritic cells from secondary lymphoid organs. Proc. Natl. Acad. Sci. USA.

[9] Mikulak J., Oriolo F., Zaghi E., Di Vito C., Mavilio D. (2017). Natural killer cells in HIV-1 infection and therapy. AIDS.

[10] Stabile H., Nisti P., Morrone S., Pagliara D., Bertaina A., Locatelli F., Santoni A., Gismondi A. (2015). Multifunctional human CD56 low CD16 low natural killer cells are the prominent subset in bone marrow of both healthy pediatric donors and leukemic patients. Haematologica.

[11] Roberto A., Di Vito C., Zaghi E., Mazza E.M.C., Capucetti A., Calvi M., Tentorio P., Zanon V., Sarina B., Mariotti J. (2018). The early expansion of anergic NKG2A(pos)/CD56(dim)/CD16(neg) natural killer represents a therapeutic target in haploidentical hematopoietic stem cell transplantation. Haematologica.

[12] Imai K., Matsuyama S., Miyake S., Suga K., Nakachi K. (2000). Natural cytotoxic activity of peripheral-blood lymphocytes and cancer incidence: An 11-year follow-up study of a general population. Lancet.

[13] Wu S.Y., Fu T., Jiang Y.Z., Shao Z.M. (2020). Natural killer cells in cancer biology and therapy. Mol. Cancer.

[14] Di Vito C., Mikulak J., Zaghi E., Pesce S., Marcenaro E., Mavilio D. (2019). NK cells to cure cancer. Semin. Immunol..

[15] Malmberg K.J., Carlsten M., Bjorklund A., Sohlberg E., Bryceson Y.T., Ljunggren H.G. (2017). Natural killer cell-mediated immunosurveillance of human cancer. Semin. Immunol..

[16] Hinshaw D.C., Shevde L.A. (2019). The Tumor Microenvironment Innately Modulates Cancer Progression. Cancer Res..

[17] Newman A.M., Liu C.L., Green M.R., Gentles A.J., Feng W., Xu Y., Hoang C.D., Diehn M., Alizadeh A.A. (2015). Robust enumeration of cell subsets from tissue expression profiles. Nat. Methods.

[18] Suen W.C., Lee W.Y., Leung K.T., Pan X.H., Li G. (2018). Natural Killer Cell-Based Cancer Immunotherapy: A Review on 10 Years Completed Clinical Trials. Cancer Investig..

[19] Collin M., Bigley V. (2018). Human dendritic cell subsets: An update. Immunology.

[20] Murphy T.L., Grajales-Reyes G.E., Wu X., Tussiwand R., Briseno C.G., Iwata A., Kretzer N.M., Durai V., Murphy K.M. (2016). Transcriptional Control of Dendritic Cell Development. Annu Rev. Immunol..

[21] Villani A.C., Satija R., Reynolds G., Sarkizova S., Shekhar K., Fletcher J., Griesbeck M., Butler A., Zheng S., Lazo S. (2017). Single-cell RNA-seq reveals new types of human blood dendritic cells, monocytes, and progenitors. Science.

[22] Robbins S.H., Walzer T., Dembele D., Thibault C., Defays A., Bessou G., Xu H., Vivier E., Sellars M., Pierre P. (2008). Novel insights into the relationships between dendritic cell subsets in human and mouse revealed by genome-wide expression profiling. Genome Biol..

[23] Bachem A., Guttler S., Hartung E., Ebstein F., Schaefer M., Tannert A., Salama A., Movassaghi K., Opitz C., Mages H.W. (2010). Superior antigen cross-presentation and XCR1 expression define human CD11c+CD141+ cells as homologues of mouse CD8+ dendritic cells. J. Exp. Med..

[24] Spranger S., Dai D., Horton B., Gajewski T.F. (2017). Tumor-Residing Batf3 Dendritic Cells Are Required for Effector T Cell Trafficking and Adoptive T Cell Therapy. Cancer Cell.

[25] Broz M.L., Binnewies M., Boldajipour B., Nelson A.E., Pollack J.L., Erle D.J., Barczak A., Rosenblum M.D., Daud A., Barber D.L. (2014). Dissecting the tumor myeloid compartment reveals rare activating antigen-presenting cells critical for T cell immunity. Cancer Cell.

[26] Bottcher J.P., Reis e Sousa C. (2018). The Role of Type 1 Conventional Dendritic Cells in Cancer Immunity. Trends Cancer.

[27] Guilliams M., Dutertre C.A., Scott C.L., McGovern N., Sichien D., Chakarov S., Van Gassen S., Chen J., Poidinger M., De Prijck S. (2016). Unsupervised High-Dimensional Analysis Aligns Dendritic Cells across Tissues and Species. Immunity.

[28] Dutertre C.A., Becht E., Irac S.E., Khalilnezhad A., Narang V., Khalilnezhad S., Ng P.Y., van den Hoogen L.L., Leong J.Y., Lee B. (2019). Single-Cell Analysis of Human Mononuclear Phagocytes Reveals Subset-Defining Markers and Identifies Circulating Inflammatory Dendritic Cells. Immunity.

[29] Alcantara-Hernandez M., Leylek R., Wagar L.E., Engleman E.G., Keler T., Marinkovich M.P., Davis M.M., Nolan G.P., Idoyaga J. (2017). High-Dimensional Phenotypic Mapping of Human Dendritic Cells Reveals Interindividual Variation and Tissue Specialization. Immunity.

[30] Anderson D.A., Murphy K.M., Briseno C.G. (2018). Development, Diversity, and Function of Dendritic Cells in Mouse and Human. Cold Spring Harb. Perspect. Biol..

[31] Yin X., Yu H., Jin X., Li J., Guo H., Shi Q., Yin Z., Xu Y., Wang X., Liu R. (2017). Human Blood CD1c+ Dendritic Cells Encompass CD5high and CD5low Subsets That Differ Significantly in Phenotype, Gene Expression, and Functions. J. Immunol..

[32] Schietinger A., Philip M., Liu R.B., Schreiber K., Schreiber H. (2010). Bystander killing of cancer requires the cooperation of CD4(+) and CD8(+) T cells during the effector phase. J. Exp. Med..

[33] Marzo A.L., Kinnear B.F., Lake R.A., Frelinger J.J., Collins E.J., Robinson B.W., Scott B. (2000). Tumor-specific CD4+ T cells have a major "post-licensing" role in CTL mediated anti-tumor immunity. J. Immunol..

[34] Zhu Z., Cuss S.M., Singh V., Gurusamy D., Shoe J.L., Leighty R., Bronte V., Hurwitz A.A. (2015). CD4+ T Cell Help Selectively Enhances High-Avidity Tumor Antigen-Specific CD8+ T Cells. J. Immunol..

[35] Gallotta M., Assi H., Degagne E., Kannan S.K., Coffman R.L., Guiducci C. (2018). Inhaled TLR9 Agonist Renders Lung Tumors Permissive to PD-1 Blockade by Promoting Optimal CD4(+) and CD8(+) T-cell Interplay. Cancer Res..

[36] Bos R., Sherman L.A. (2010). CD4+ T-cell help in the tumor milieu is required for recruitment and cytolytic function of CD8+ T lymphocytes. Cancer Res..

[37] Labidi-Galy S.I., Treilleux I., Goddard-Leon S., Combes J.D., Blay J.Y., Ray-Coquard I., Caux C., Bendriss-Vermare N. (2012). Plasmacytoid dendritic cells infiltrating ovarian cancer are associated with poor prognosis. Oncoimmunology.

[38] Aspord C., Leccia M.T., Charles J., Plumas J. (2013). Plasmacytoid dendritic cells support melanoma progression by promoting Th2 and regulatory immunity through OX40L and ICOSL. Cancer Immunol. Res..

[39] Treilleux I., Blay J.Y., Bendriss-Vermare N., Ray-Coquard I., Bachelot T., Guastalla J.P., Bremond A., Goddard S., Pin J.J., Barthelemy-Dubois C. (2004). Dendritic cell infiltration and prognosis of early stage breast cancer. Clin. Cancer Res..

[40] Conrad C., Gregorio J., Wang Y.H., Ito T., Meller S., Hanabuchi S., Anderson S., Atkinson N., Ramirez P.T., Liu Y.J. (2012). Plasmacytoid dendritic cells promote immunosuppression in ovarian cancer via ICOS costimulation of Foxp3(+) T-regulatory cells. Cancer Res..

[41] Tel J., Schreibelt G., Sittig S.P., Mathan T.S., Buschow S.I., Cruz L.J., Lambeck A.J., Figdor C.G., de Vries I.J. (2013). Human plasmacytoid dendritic cells efficiently cross-present exogenous Ags to CD8+ T cells despite lower Ag uptake than myeloid dendritic cell subsets. Blood.

[42] Colonna M., Cella M. (2007). Crosspresentation: Plasmacytoid dendritic cells are in the business. Immunity.

[43] Villadangos J.A., Young L. (2008). Antigen-presentation properties of plasmacytoid dendritic cells. Immunity.

[44] Nierkens S., Tel J., Janssen E., Adema G.J. (2013). Antigen cross-presentation by dendritic cell subsets: One general or all sergeants?. Trends Immunol..

[45] Lou Y., Liu C., Kim G.J., Liu Y.J., Hwu P., Wang G. (2007). Plasmacytoid dendritic cells synergize with myeloid dendritic cells in the induction of antigen-specific antitumor immune responses. J. Immunol..

[46] Fu C., Peng P., Loschko J., Feng L., Pham P., Cui W., Lee K.P., Krug A.B., Jiang A. (2020). Plasmacytoid dendritic cells cross-prime naive CD8 T cells by transferring antigen to conventional dendritic cells through exosomes. Proc. Natl. Acad. Sci. USA.

[47] Noubade R., Majri-Morrison S., Tarbell K.V. (2019). Beyond cDC1: Emerging Roles of DC Crosstalk in Cancer Immunity. Front. Immunol..

[48] Dieu-Nosjean M.C., Giraldo N.A., Kaplon H., Germain C., Fridman W.H., Sautes-Fridman C. (2016). Tertiary lymphoid structures, drivers of the anti-tumor responses in human cancers. Immunol. Rev..

[49] Binnewies M., Roberts E.W., Kersten K., Chan V., Fearon D.F., Merad M., Coussens L.M., Gabrilovich D.I., Ostrand-Rosenberg S., Hedrick C.C. (2018). Understanding the tumor immune microenvironment (TIME) for effective therapy. Nat. Med..

[50] Carenza C., Franzese S., Calcaterra F., Mavilio D., Della Bella S. (2020). Comprehensive Phenotyping of Dendritic Cells in Cancer Patients by Flow Cytometry. Cytom. A.

[51] Mittal D., Gubin M.M., Schreiber R.D., Smyth M.J. (2014). New insights into cancer immunoediting and its three component phases--elimination, equilibrium and escape. Curr. Opin. Immunol..

[52] Schreiber R.D., Old L.J., Smyth M.J. (2011). Cancer immunoediting: Integrating immunity’s roles in cancer suppression and promotion. Science.

[53] Tran Janco J.M., Lamichhane P., Karyampudi L., Knutson K.L. (2015). Tumor-infiltrating dendritic cells in cancer pathogenesis. J. Immunol..

[54] Della Bella S., Clerici M., Villa M.L. (2007). Disarming dendritic cells: A tumor strategy to escape from immune control?. Expert Rev. Clin. Immunol..

[55] Carenza C., Calcaterra F., Oriolo F., Di Vito C., Ubezio M., Della Porta M.G., Mavilio D., Della Bella S. (2019). Costimulatory Molecules and Immune Checkpoints Are Differentially Expressed on Different Subsets of Dendritic Cells. Front. Immunol..

[56] Della Bella S., Nicola S., Brambilla L., Riva A., Ferrucci S., Presicce P., Boneschi V., Berti E., Villa M.L. (2006). Quantitative and functional defects of dendritic cells in classic Kaposi’s sarcoma. Clin. Immunol..

[57] Della Bella S., Gennaro M., Vaccari M., Ferraris C., Nicola S., Riva A., Clerici M., Greco M., Villa M.L. (2003). Altered maturation of peripheral blood dendritic cells in patients with breast cancer. Br. J. Cancer.

[58] Fernandez N.C., Lozier A., Flament C., Ricciardi-Castagnoli P., Bellet D., Suter M., Perricaudet M., Tursz T., Maraskovsky E., Zitvogel L. (1999). Dendritic cells directly trigger NK cell functions: Cross-talk relevant in innate anti-tumor immune responses in vivo. Nat. Med..

[59] Thomas R., Yang X. (2016). NK-DC Crosstalk in Immunity to Microbial Infection. J. Immunol. Res..

[60] Guillerey C., Huntington N.D., Smyth M.J. (2016). Targeting natural killer cells in cancer immunotherapy. Nat. Immunol..

[61] Dalod M., Hamilton T., Salomon R., Salazar-Mather T.P., Henry S.C., Hamilton J.D., Biron C.A. (2003). Dendritic cell responses to early murine cytomegalovirus infection: Subset functional specialization and differential regulation by interferon alpha/beta. J. Exp. Med..

[62] Krug A., French A.R., Barchet W., Fischer J.A., Dzionek A., Pingel J.T., Orihuela M.M., Akira S., Yokoyama W.M., Colonna M. (2004). TLR9-dependent recognition of MCMV by IPC and DC generates coordinated cytokine responses that activate antiviral NK cell function. Immunity.

[63] Kassim S.H., Rajasagi N.K., Ritz B.W., Pruett S.B., Gardner E.M., Chervenak R., Jennings S.R. (2009). Dendritic cells are required for optimal activation of natural killer functions following primary infection with herpes simplex virus type 1. J. Virol.

[64] Barr D.P., Belz G.T., Reading P.C., Wojtasiak M., Whitney P.G., Heath W.R., Carbone F.R., Brooks A.G. (2007). A role for plasmacytoid dendritic cells in the rapid IL-18-dependent activation of NK cells following HSV-1 infection. Eur J. Immunol..

[65] Vogel K., Thomann S., Vogel B., Schuster P., Schmidt B. (2014). Both plasmacytoid dendritic cells and monocytes stimulate natural killer cells early during human herpes simplex virus type 1 infections. Immunology.

[66] Van den Broeke L.T., Daschbach E., Thomas E.K., Andringa G., Berzofsky J.A. (2003). Dendritic cell-induced activation of adaptive and innate antitumor immunity. J. Immunol..

[67] Mingozzi F., Spreafico R., Gorletta T., Cigni C., Di Gioia M., Caccia M., Sironi L., Collini M., Soncini M., Rusconi M. (2016). Prolonged contact with dendritic cells turns lymph node-resident NK cells into anti-tumor effectors. EMBO Mol. Med..

[68] Lucas M., Schachterle W., Oberle K., Aichele P., Diefenbach A. (2007). Dendritic cells prime natural killer cells by trans-presenting interleukin 15. Immunity.

[69] Brilot F., Strowig T., Roberts S.M., Arrey F., Munz C. (2007). NK cell survival mediated through the regulatory synapse with human DCs requires IL-15Ralpha. J. Clin. Invest..

[70] Vujanovic L., Szymkowski D.E., Alber S., Watkins S.C., Vujanovic N.L., Butterfield L.H. (2010). Virally infected and matured human dendritic cells activate natural killer cells via cooperative activity of plasma membrane-bound TNF and IL-15. Blood.

[71] Zanoni I., Spreafico R., Bodio C., Di Gioia M., Cigni C., Broggi A., Gorletta T., Caccia M., Chirico G., Sironi L. (2013). IL-15 cis presentation is required for optimal NK cell activation in lipopolysaccharide-mediated inflammatory conditions. Cell Rep..

[72] Benlahrech A., Donaghy H., Rozis G., Goodier M., Klavinskis L., Gotch F., Patterson S. (2009). Human NK Cell Up-regulation of CD69, HLA-DR, Interferon gamma Secretion and Cytotoxic Activity by Plasmacytoid Dendritic Cells is Regulated through Overlapping but Different Pathways. Sensors.

[73] Marshall J.D., Heeke D.S., Abbate C., Yee P., Van Nest G. (2006). Induction of interferon-gamma from natural killer cells by immunostimulatory CpG DNA is mediated through plasmacytoid-dendritic-cell-produced interferon-alpha and tumour necrosis factor-alpha. Immunology.

[74] Gerosa F., Gobbi A., Zorzi P., Burg S., Briere F., Carra G., Trinchieri G. (2005). The reciprocal interaction of NK cells with plasmacytoid or myeloid dendritic cells profoundly affects innate resistance functions. J. Immunol..

[75] Jinushi M., Takehara T., Kanto T., Tatsumi T., Groh V., Spies T., Miyagi T., Suzuki T., Sasaki Y., Hayashi N. (2003). Critical role of MHC class I-related chain A and B expression on IFN-alpha-stimulated dendritic cells in NK cell activation: Impairment in chronic hepatitis C virus infection. J. Immunol..

[76] Draghi M., Pashine A., Sanjanwala B., Gendzekhadze K., Cantoni C., Cosman D., Moretta A., Valiante N.M., Parham P. (2007). NKp46 and NKG2D recognition of infected dendritic cells is necessary for NK cell activation in the human response to influenza infection. J. Immunol..

[77] Hanabuchi S., Watanabe N., Wang Y.H., Wang Y.H., Ito T., Shaw J., Cao W., Qin F.X., Liu Y.J. (2006). Human plasmacytoid predendritic cells activate NK cells through glucocorticoid-induced tumor necrosis factor receptor-ligand (GITRL). Blood.

[78] Borg C., Jalil A., Laderach D., Maruyama K., Wakasugi H., Charrier S., Ryffel B., Cambi A., Figdor C., Vainchenker W. (2004). NK cell activation by dendritic cells (DCs) requires the formation of a synapse leading to IL-12 polarization in DCs. Blood.

[79] Nishioka Y., Nishimura N., Suzuki Y., Sone S. (2001). Human monocyte-derived and CD83(+) blood dendritic cells enhance NK cell-mediated cytotoxicity. Eur. J. Immunol..

[80] Schmitz M., Zhao S., Deuse Y., Schakel K., Wehner R., Wohner H., Holig K., Wienforth F., Kiessling A., Bornhauser M. (2005). Tumoricidal potential of native blood dendritic cells: Direct tumor cell killing and activation of NK cell-mediated cytotoxicity. J. Immunol..

[81] Wehner R., Lobel B., Bornhauser M., Schakel K., Cartellieri M., Bachmann M., Rieber E.P., Schmitz M. (2009). Reciprocal activating interaction between 6-sulfo LacNAc+ dendritic cells and NK cells. Int J. Cancer.

[82] Wilson J.L., Heffler L.C., Charo J., Scheynius A., Bejarano M.T., Ljunggren H.G. (1999). Targeting of human dendritic cells by autologous NK cells. J. Immunol..

[83] Ferlazzo G., Tsang M.L., Moretta L., Melioli G., Steinman R.M., Munz C. (2002). Human dendritic cells activate resting natural killer (NK) cells and are recognized via the NKp30 receptor by activated NK cells. J. Exp. Med..

[84] Castriconi R., Cantoni C., Della Chiesa M., Vitale M., Marcenaro E., Conte R., Biassoni R., Bottino C., Moretta L., Moretta A. (2003). Transforming growth factor beta 1 inhibits expression of NKp30 and NKG2D receptors: Consequences for the NK-mediated killing of dendritic cells. Proc. Natl. Acad. Sci. USA.

[85] Della Chiesa M., Vitale M., Carlomagno S., Ferlazzo G., Moretta L., Moretta A. (2003). The natural killer cell-mediated killing of autologous dendritic cells is confined to a cell subset expressing CD94/NKG2A, but lacking inhibitory killer Ig-like receptors. Eur. J. Immunol..

[86] Pende D., Castriconi R., Romagnani P., Spaggiari G.M., Marcenaro S., Dondero A., Lazzeri E., Lasagni L., Martini S., Rivera P. (2006). Expression of the DNAM-1 ligands, Nectin-2 (CD112) and poliovirus receptor (CD155), on dendritic cells: Relevance for natural killer-dendritic cell interaction. Blood.

[87] Smith L.E., Olszewski M.A., Georgoudaki A.M., Wagner A.K., Hagglof T., Karlsson M.C., Dominguez-Villar M., Garcia-Cozar F., Mueller S., Ravens I. (2016). Sensitivity of dendritic cells to NK-mediated lysis depends on the inflammatory environment and is modulated by CD54/CD226-driven interactions. J. Leukoc. Biol..

[88] Walwyn-Brown K., Guldevall K., Saeed M., Pende D., Onfelt B., MacDonald A.S., Davis D.M. (2018). Human NK Cells Lyse Th2-Polarizing Dendritic Cells via NKp30 and DNAM-1. J. Immunol..

[89] Piccioli D., Sbrana S., Melandri E., Valiante N.M. (2002). Contact-dependent stimulation and inhibition of dendritic cells by natural killer cells. J. Exp. Med..

[90] Vitale M., Della Chiesa M., Carlomagno S., Pende D., Arico M., Moretta L., Moretta A. (2005). NK-dependent DC maturation is mediated by TNFalpha and IFNgamma released upon engagement of the NKp30 triggering receptor. Blood.

[91] Mailliard R.B., Son Y.I., Redlinger R., Coates P.T., Giermasz A., Morel P.A., Storkus W.J., Kalinski P. (2003). Dendritic cells mediate NK cell help for Th1 and CTL responses: Two-signal requirement for the induction of NK cell helper function. J. Immunol..

[92] Agaugue S., Marcenaro E., Ferranti B., Moretta L., Moretta A. (2008). Human natural killer cells exposed to IL-2, IL-12, IL-18, or IL-4 differently modulate priming of naive T cells by monocyte-derived dendritic cells. Blood.

[93] Marcenaro E., Della Chiesa M., Bellora F., Parolini S., Millo R., Moretta L., Moretta A. (2005). IL-12 or IL-4 prime human NK cells to mediate functionally divergent interactions with dendritic cells or tumors. J. Immunol..

[94] Clavijo-Salomon M.A., Salcedo R., Roy S., das Neves R.X., Dzutsev A., Sales-Campos H., Borbely K.S., Silla L., Orange J.S., Mace E.M. (2020). Human NK cells prime inflammatory DC precursors to induce Tc17 differentiation. Blood Adv..

[95] Carrega P., Bonaccorsi I., Di Carlo E., Morandi B., Paul P., Rizzello V., Cipollone G., Navarra G., Mingari M.C., Moretta L. (2014). CD56(bright)perforin(low) noncytotoxic human NK cells are abundant in both healthy and neoplastic solid tissues and recirculate to secondary lymphoid organs via afferent lymph. J. Immunol..

[96] Freud A.G., Mundy-Bosse B.L., Yu J., Caligiuri M.A. (2017). The Broad Spectrum of Human Natural Killer Cell Diversity. Immunity.

[97] Mikulak J., Bruni E., Oriolo F., Di Vito C., Mavilio D. (2019). Hepatic Natural Killer Cells: Organ-Specific Sentinels of Liver Immune Homeostasis and Physiopathology. Front. Immunol..

[98] Hudspeth K., Pontarini E., Tentorio P., Cimino M., Donadon M., Torzilli G., Lugli E., Della Bella S., Gershwin M.E., Mavilio D. (2013). The role of natural killer cells in autoimmune liver disease: A comprehensive review. J. Autoimmun..

[99] Hudspeth K., Donadon M., Cimino M., Pontarini E., Tentorio P., Preti M., Hong M., Bertoletti A., Bicciato S., Invernizzi P. (2016). Human liver-resident CD56(bright)/CD16(neg) NK cells are retained within hepatic sinusoids via the engagement of CCR5 and CXCR6 pathways. J. Autoimmun..

[100] Luo D.Z., Vermijlen D., Ahishali B., Triantis V., Plakoutsi G., Braet F., Vanderkerken K., Wisse E. (2000). On the cell biology of pit cells, the liver-specific NK cells. World J. Gastroenterol..

[101] Doherty D.G., Norris S., Madrigal-Estebas L., McEntee G., Traynor O., Hegarty J.E., O’Farrelly C. (1999). The human liver contains multiple populations of NK cells, T cells, and CD3+CD56+ natural T cells with distinct cytotoxic activities and Th1, Th2, and Th0 cytokine secretion patterns. J. Immunol..

[102] Stegmann K.A., Robertson F., Hansi N., Gill U., Pallant C., Christophides T., Pallett L.J., Peppa D., Dunn C., Fusai G. (2016). CXCR6 marks a novel subset of T-bet(lo)Eomes(hi) natural killer cells residing in human liver. Sci Rep..

[103] Marquardt N., Beziat V., Nystrom S., Hengst J., Ivarsson M.A., Kekalainen E., Johansson H., Mjosberg J., Westgren M., Lankisch T.O. (2015). Cutting edge: Identification and characterization of human intrahepatic CD49a+ NK cells. J. Immunol..

[104] Mikulak J., Di Vito C., Zaghi E., Mavilio D. (2017). Host Immune Responses in HIV-1 Infection: The Emerging Pathogenic Role of Siglecs and Their Clinical Correlates. Front. Immunol..

[105] Lalor P.F., Edwards S., McNab G., Salmi M., Jalkanen S., Adams D.H. (2002). Vascular adhesion protein-1 mediates adhesion and transmigration of lymphocytes on human hepatic endothelial cells. J. Immunol..

[106] Harmon C., Robinson M.W., Fahey R., Whelan S., Houlihan D.D., Geoghegan J., O’Farrelly C. (2016). Tissue-resident Eomes(hi) T-bet(lo) CD56(bright) NK cells with reduced proinflammatory potential are enriched in the adult human liver. Eur. J. Immunol..

[107] Cuff A.O., Robertson F.P., Stegmann K.A., Pallett L.J., Maini M.K., Davidson B.R., Male V. (2016). Eomeshi NK Cells in Human Liver Are Long-Lived and Do Not Recirculate but Can Be Replenished from the Circulation. J. Immunol..

[108] Cerwenka A., Lanier L.L. (2016). Natural killer cell memory in infection, inflammation and cancer. Nat. Rev. Immunol..

[109] Lopez-Verges S., Milush J.M., Schwartz B.S., Pando M.J., Jarjoura J., York V.A., Houchins J.P., Miller S., Kang S.M., Norris P.J. (2011). Expansion of a unique CD57(+)NKG2Chi natural killer cell subset during acute human cytomegalovirus infection. Proc. Natl. Acad. Sci. USA.

[110] Reeves R.K., Li H., Jost S., Blass E., Li H., Schafer J.L., Varner V., Manickam C., Eslamizar L., Altfeld M. (2015). Antigen-specific NK cell memory in rhesus macaques. Nat. Immunol..

[111] Hydes T., Abuhilal M., Armstrong T., Primrose J., Takhar A., Khakoo S. (2015). Natural killer cell maturation markers in the human liver and expansion of an NKG2C+KIR+ population. Lancet.

[112] Schlums H., Cichocki F., Tesi B., Theorell J., Beziat V., Holmes T.D., Han H., Chiang S.C., Foley B., Mattsson K. (2015). Cytomegalovirus infection drives adaptive epigenetic diversification of NK cells with altered signaling and effector function. Immunity.

[113] Lee J., Zhang T., Hwang I., Kim A., Nitschke L., Kim M., Scott J.M., Kamimura Y., Lanier L.L., Kim S. (2015). Epigenetic modification and antibody-dependent expansion of memory-like NK cells in human cytomegalovirus-infected individuals. Immunity.

[114] Zhao J., Zhang S., Liu Y., He X., Qu M., Xu G., Wang H., Huang M., Pan J., Liu Z. (2020). Single-cell RNA sequencing reveals the heterogeneity of liver-resident immune cells in human. Cell Discov..

[115] MacParland S.A., Liu J.C., Ma X.Z., Innes B.T., Bartczak A.M., Gage B.K., Manuel J., Khuu N., Echeverri J., Linares I. (2018). Single cell RNA sequencing of human liver reveals distinct intrahepatic macrophage populations. Nat. Commun..

[116] Filipovic I., Sonnerborg I., Strunz B., Friberg D., Cornillet M., Hertwig L., Ivarsson M.A., Bjorkstrom N.K. (2019). 29-Color Flow Cytometry: Unraveling Human Liver NK Cell Repertoire Diversity. Front. Immunol..

[117] Cunningham E.C., Sharland A.F., Bishop G.A. (2013). Liver transplant tolerance and its application to the clinic: Can we exploit the high dose effect?. Clin. Dev. Immunol..

[118] Wang Y., Zhang C. (2019). The Roles of Liver-Resident Lymphocytes in Liver Diseases. Front. Immunol..

[119] Crispe I.N. (2014). Immune tolerance in liver disease. Hepatology.

[120] Lassen M.G., Lukens J.R., Dolina J.S., Brown M.G., Hahn Y.S. (2010). Intrahepatic IL-10 maintains NKG2A+Ly49- liver NK cells in a functionally hyporesponsive state. J. Immunol..

[121] Tu Z., Bozorgzadeh A., Pierce R.H., Kurtis J., Crispe I.N., Orloff M.S. (2008). TLR-dependent cross talk between human Kupffer cells and NK cells. J. Exp. Med..

[122] Jinushi M., Takehara T., Tatsumi T., Yamaguchi S., Sakamori R., Hiramatsu N., Kanto T., Ohkawa K., Hayashi N. (2007). Natural killer cell and hepatic cell interaction via NKG2A leads to dendritic cell-mediated induction of CD4 CD25 T cells with PD-1-dependent regulatory activities. Immunology.

[123] Chong W.P., Zhou J., Law H.K., Tu W., Lau Y.L. (2010). Natural killer cells become tolerogenic after interaction with apoptotic cells. Eur. J. Immunol..

[124] Ishiyama K., Ohdan H., Ohira M., Mitsuta H., Arihiro K., Asahara T. (2006). Difference in cytotoxicity against hepatocellular carcinoma between liver and periphery natural killer cells in humans. Hepatology.

[125] Li N., Puga Yung G.L., Pradier A., Toso C., Giostra E., Morard I., Spahr L., Seebach J.D. (2013). NK cell isolation from liver biopsies: Phenotypic and functional analysis of low cell numbers by flow cytometry. Front. Immunol..

[126] Tang L., Peng H., Zhou J., Chen Y., Wei H., Sun R., Yokoyama W.M., Tian Z. (2016). Differential phenotypic and functional properties of liver-resident NK cells and mucosal ILC1s. J. Autoimmun..

[127] Sojka D.K., Plougastel-Douglas B., Yang L., Pak-Wittel M.A., Artyomov M.N., Ivanova Y., Zhong C., Chase J.M., Rothman P.B., Yu J. (2014). Tissue-resident natural killer (NK) cells are cell lineages distinct from thymic and conventional splenic NK cells. eLife.

[128] Daussy C., Faure F., Mayol K., Viel S., Gasteiger G., Charrier E., Bienvenu J., Henry T., Debien E., Hasan U.A. (2014). T-bet and Eomes instruct the development of two distinct natural killer cell lineages in the liver and in the bone marrow. J. Exp. Med..

[129] Amadei B., Urbani S., Cazaly A., Fisicaro P., Zerbini A., Ahmed P., Missale G., Ferrari C., Khakoo S.I. (2010). Activation of natural killer cells during acute infection with hepatitis C virus. Gastroenterology.

[130] Fisicaro P., Valdatta C., Boni C., Massari M., Mori C., Zerbini A., Orlandini A., Sacchelli L., Missale G., Ferrari C. (2009). Early kinetics of innate and adaptive immune responses during hepatitis B virus infection. Gut.

[131] Li J., Han Y., Jin K., Wan Y., Wang S., Liu B., Liu Y., Lu S., Huang Z. (2011). Dynamic changes of cytotoxic T lymphocytes (CTLs), natural killer (NK) cells, and natural killer T (NKT) cells in patients with acute hepatitis B infection. Virol. J..

[132] Graubardt N., Fahrner R., Trochsler M., Keogh A., Breu K., Furer C., Stroka D., Robson S.C., Slack E., Candinas D. (2013). Promotion of liver regeneration by natural killer cells in a murine model is dependent on extracellular adenosine triphosphate phosphohydrolysis. Hepatology.

[133] Radaeva S., Sun R., Jaruga B., Nguyen V.T., Tian Z., Gao B. (2006). Natural killer cells ameliorate liver fibrosis by killing activated stellate cells in NKG2D-dependent and tumor necrosis factor-related apoptosis-inducing ligand-dependent manners. Gastroenterology.

[134] Wang J., Sun R., Wei H., Dong Z., Gao B., Tian Z. (2006). Poly I:C prevents T cell-mediated hepatitis via an NK-dependent mechanism. J. Hepatol..

[135] Tosello-Trampont A., Surette F.A., Ewald S.E., Hahn Y.S. (2017). Immunoregulatory Role of NK Cells in Tissue Inflammation and Regeneration. Front. Immunol..

[136] Thomson A.W., Knolle P.A. (2010). Antigen-presenting cell function in the tolerogenic liver environment. Nat. Rev. Immunol..

[137] Bosma B.M., Metselaar H.J., Mancham S., Boor P.P., Kusters J.G., Kazemier G., Tilanus H.W., Kuipers E.J., Kwekkeboom J. (2006). Characterization of human liver dendritic cells in liver grafts and perfusates. Liver Transpl..

[138] Strauss O., Dunbar P.R., Bartlett A., Phillips A. (2015). The immunophenotype of antigen presenting cells of the mononuclear phagocyte system in normal human liver--A systematic review. J. Hepatol..

[139] Crosignani A., Riva A., Della Bella S. (2016). Analysis of peripheral blood dendritic cells as a non-invasive tool in the follow-up of patients with chronic hepatitis C. World J. Gastroenterol..

[140] Bamboat Z.M., Stableford J.A., Plitas G., Burt B.M., Nguyen H.M., Welles A.P., Gonen M., Young J.W., DeMatteo R.P. (2009). Human liver dendritic cells promote T cell hyporesponsiveness. J. Immunol..

[141] Xia S., Guo Z., Xu X., Yi H., Wang Q., Cao X. (2008). Hepatic microenvironment programs hematopoietic progenitor differentiation into regulatory dendritic cells, maintaining liver tolerance. Blood.

[142] Kelly A., Fahey R., Fletcher J.M., Keogh C., Carroll A.G., Siddachari R., Geoghegan J., Hegarty J.E., Ryan E.J., O’Farrelly C. (2014). CD141(+) myeloid dendritic cells are enriched in healthy human liver. J. Hepatol..

[143] Ibrahim J., Nguyen A.H., Rehman A., Ochi A., Jamal M., Graffeo C.S., Henning J.R., Zambirinis C.P., Fallon N.C., Barilla R. (2012). Dendritic cell populations with different concentrations of lipid regulate tolerance and immunity in mouse and human liver. Gastroenterology.

[144] Cantoni C., Huergo-Zapico L., Parodi M., Pedrazzi M., Mingari M.C., Moretta A., Sparatore B., Gonzalez S., Olive D., Bottino C. (2016). NK Cells, Tumor Cell Transition, and Tumor Progression in Solid Malignancies: New Hints for NK-Based Immunotherapy?. J. Immunol. Res..

[145] Chiossone L., Vienne M., Kerdiles Y.M., Vivier E. (2017). Natural killer cell immunotherapies against cancer: Checkpoint inhibitors and more. Semin. Immunol..

[146] Chew V., Tow C., Teo M., Wong H.L., Chan J., Gehring A., Loh M., Bolze A., Quek R., Lee V.K. (2010). Inflammatory tumour microenvironment is associated with superior survival in hepatocellular carcinoma patients. J. Hepatol..

[147] Chew V., Chen J., Lee D., Loh E., Lee J., Lim K.H., Weber A., Slankamenac K., Poon R.T., Yang H. (2012). Chemokine-driven lymphocyte infiltration: An early intratumoural event determining long-term survival in resectable hepatocellular carcinoma. Gut.

[148] Cai L., Zhang Z., Zhou L., Wang H., Fu J., Zhang S., Shi M., Zhang H., Yang Y., Wu H. (2008). Functional impairment in circulating and intrahepatic NK cells and relative mechanism in hepatocellular carcinoma patients. Clin. Immunol..

[149] Abdelrahman M.M., Fawzy I.O., Bassiouni A.A., Gomaa A.I., Esmat G., Waked I., Abdelaziz A.I. (2016). Enhancing NK cell cytotoxicity by miR-182 in hepatocellular carcinoma. Hum. Immunol..

[150] Lasfar A., de laTorre A., Abushahba W., Cohen-Solal K.A., Castaneda I., Yuan Y., Reuhl K., Zloza A., Raveche E., Laskin D.L. (2016). Concerted action of IFN-alpha and IFN-lambda induces local NK cell immunity and halts cancer growth. Oncotarget.

[151] Fathy A., Eldin M.M., Metwally L., Eida M., Abdel-Rehim M. (2009). Diminished absolute counts of CD56dim and CD56bright natural killer cells in peripheral blood from Egyptian patients with hepatocellular carcinoma. Egypt J. Immunol..

[152] Wu Y., Kuang D.M., Pan W.D., Wan Y.L., Lao X.M., Wang D., Li X.F., Zheng L. (2013). Monocyte/macrophage-elicited natural killer cell dysfunction in hepatocellular carcinoma is mediated by CD48/2B4 interactions. Hepatology.

[153] Liu Y., Cheng Y., Xu Y., Wang Z., Du X., Li C., Peng J., Gao L., Liang X., Ma C. (2017). Increased expression of programmed cell death protein 1 on NK cells inhibits NK-cell-mediated anti-tumor function and indicates poor prognosis in digestive cancers. Oncogene.

[154] Sun C., Xu J., Huang Q., Huang M., Wen H., Zhang C., Wang J., Song J., Zheng M., Sun H. (2017). High NKG2A expression contributes to NK cell exhaustion and predicts a poor prognosis of patients with liver cancer. Oncoimmunology.

[155] Huang J., Cai M.Y., Wei D.P. (2002). HLA class I expression in primary hepatocellular carcinoma. World J. Gastroenterol..

[156] Kudo M. (2017). Immuno-Oncology in Hepatocellular Carcinoma: 2017 Update. Oncology.

[157] Mantovani S., Oliviero B., Lombardi A., Varchetta S., Mele D., Sangiovanni A., Rossi G., Donadon M., Torzilli G., Soldani C. (2018). Deficient natural killer cell NKp30-mediated function and altered NCR3 splice variants in hepatocellular carcinoma. Hepatology.

[158] Hoechst B., Voigtlaender T., Ormandy L., Gamrekelashvili J., Zhao F., Wedemeyer H., Lehner F., Manns M.P., Greten T.F., Korangy F. (2009). Myeloid derived suppressor cells inhibit natural killer cells in patients with hepatocellular carcinoma via the NKp30 receptor. Hepatology.

[159] Polidoro M.A., Mikulak J., Cazzetta V., Lleo A., Mavilio D., Torzilli G., Donadon M. (2020). Tumor microenvironment in primary liver tumors: A challenging role of natural killer cells. World J. Gastroenterol.

[160] Wallace M.C., Preen D., Jeffrey G.P., Adams L.A. (2015). The evolving epidemiology of hepatocellular carcinoma: A global perspective. Expert Rev. Gastroenterol. Hepatol..

[161] Rehermann B. (2015). Natural Killer Cells in Viral Hepatitis. Cell Mol. Gastroenterol. Hepatol..

[162] Wu S.F., Wang W.J., Gao Y.Q. (2015). Natural killer cells in hepatitis B virus infection. Braz J. Infect. Dis.

[163] Oliviero B., Varchetta S., Paudice E., Michelone G., Zaramella M., Mavilio D., De Filippi F., Bruno S., Mondelli M.U. (2009). Natural killer cell functional dichotomy in chronic hepatitis B and chronic hepatitis C virus infections. Gastroenterology.

[164] Tjwa E.T., van Oord G.W., Hegmans J.P., Janssen H.L., Woltman A.M. (2011). Viral load reduction improves activation and function of natural killer cells in patients with chronic hepatitis B. J. Hepatol..

[165] Miyagi T., Gil M.P., Wang X., Louten J., Chu W.M., Biron C.A. (2007). High basal STAT4 balanced by STAT1 induction to control type 1 interferon effects in natural killer cells. J. Exp. Med..

[166] Peppa D., Gill U.S., Reynolds G., Easom N.J., Pallett L.J., Schurich A., Micco L., Nebbia G., Singh H.D., Adams D.H. (2013). Up-regulation of a death receptor renders antiviral T cells susceptible to NK cell-mediated deletion. J. Exp. Med..

[167] Tatsumi T., Takehara T. (2016). Impact of natural killer cells on chronic hepatitis C and hepatocellular carcinoma. Hepatol. Res..

[168] Morisaki T., Umebayashi M., Kiyota A., Koya N., Tanaka H., Onishi H., Katano M. (2012). Combining cetuximab with killer lymphocytes synergistically inhibits human cholangiocarcinoma cells in vitro. Anticancer Res..

[169] Jung I.H., Kim D.H., Yoo D.K., Baek S.Y., Jeong S.H., Jung D.E., Park S.W., Chung Y.Y. (2018). In Vivo Study of Natural Killer (NK) Cell Cytotoxicity Against Cholangiocarcinoma in a Nude Mouse Model. In Vivo.

[170] Fukuda Y., Asaoka T., Eguchi H., Yokota Y., Kubo M., Kinoshita M., Urakawa S., Iwagami Y., Tomimaru Y., Akita H. (2020). Endogenous CXCL9 affects prognosis by regulating tumor-infiltrating natural killer cells in intrahepatic cholangiocarcinoma. Cancer Sci..

[171] Tsukagoshi M., Wada S., Yokobori T., Altan B., Ishii N., Watanabe A., Kubo N., Saito F., Araki K., Suzuki H. (2016). Overexpression of natural killer group 2 member D ligands predicts favorable prognosis in cholangiocarcinoma. Cancer Sci..

[172] Carnevale G., Carpino G., Cardinale V., Pisciotta A., Riccio M., Bertoni L., Gibellini L., De Biasi S., Nevi L., Costantini D. (2017). Activation of Fas/FasL pathway and the role of c-FLIP in primary culture of human cholangiocarcinoma cells. Sci Rep..

[173] Hayashi T., Imai K., Morishita Y., Hayashi I., Kusunoki Y., Nakachi K. (2006). Identification of the NKG2D haplotypes associated with natural cytotoxic activity of peripheral blood lymphocytes and cancer immunosurveillance. Cancer Res..

[174] Chen S.R., Akbar S.M.F., Tanimoto K., Ninomiya T., Iuchi H., Michitaka K., Horiike N., Onji M. (2000). Absence of CD83-positive mature and activated dendritic cells at cancer nodules from patients with hepatocellular carcinoma: Relevance to hepatocarcinogenesis. Cancer Lett..

[175] Tang T.J., Vukosavljevic D., Janssen H.L., Binda R.S., Mancham S., Tilanus H.W., Ijzermans J.N., Drexhage H., Kwekkeboom J. (2006). Aberrant composition of the dendritic cell population in hepatic lymph nodes of patients with hepatocellular carcinoma. Hum. Pathol..

[176] Pedroza-Gonzalez A., Zhou G., Vargas-Mendez E., Boor P.P., Mancham S., Verhoef C., Polak W.G., Grunhagen D., Pan Q., Janssen H. (2015). Tumor-infiltrating plasmacytoid dendritic cells promote immunosuppression by Tr1 cells in human liver tumors. OncoImmunology.

[177] Kunitani H., Shimizu Y., Murata H., Higuchi K., Watanabe A. (2002). Phenotypic analysis of circulating and intrahepatic dendritic cell subsets in patients with chronic liver diseases. J. Hepatol..

[178] Beckebaum S., Zhang X., Chen X., Yu Z., Frilling A., Dworacki G., Grosse-Wilde H., Broelsch C.E., Gerken G., Cicinnati V.R. (2004). Increased levels of interleukin-10 in serum from patients with hepatocellular carcinoma correlate with profound numerical deficiencies and immature phenotype of circulating dendritic cell subsets. Clin. Cancer Res..

[179] Martin-Sierra C., Martins R., Laranjeira P., Abrantes A.M., Oliveira R.C., Tralhao J.G., Botelho M.F., Furtado E., Domingues R., Paiva A. (2019). Functional Impairment of Circulating FcepsilonRI(+) Monocytes and Myeloid Dendritic Cells in Hepatocellular Carcinoma and Cholangiocarcinoma Patients. Cytom. B Clin. Cytom..

[180] Ritter M., Ali M.Y., Grimm C.F., Weth R., Mohr L., Bocher W.O., Endrulat K., Wedemeyer H., Blum H.E., Geissler M. (2004). Immunoregulation of dendritic and T cells by alpha-fetoprotein in patients with hepatocellular carcinoma. J. Hepatol..

[181] Ninomiya T., Akbar F., Masumoto T., Horiike N., Onji M. (1999). Dendritic cells with immature phenotype and defective function in the peripheral blood from patients with hepatocellular carcinoma. J. Hepatol..

[182] Kakumu S., Ito S., Ishikawa T., Mita Y., Tagaya T., Fukuzawa Y., Yoshioka K. (2000). Decreased function of peripheral blood dendritic cells in patients with hepatocellular carcinoma with hepatitis B and C virus infection. J. Gastroenterol. Hepatol..

[183] Zhang Q., He Y., Luo N., Patel S.J., Han Y., Gao R., Modak M., Carotta S., Haslinger C., Kind D. (2019). Landscape and Dynamics of Single Immune Cells in Hepatocellular Carcinoma. Cell.

[184] Michea P., Noel F., Zakine E., Czerwinska U., Sirven P., Abouzid O., Goudot C., Scholer-Dahirel A., Vincent-Salomon A., Reyal F. (2018). Adjustment of dendritic cells to the breast-cancer microenvironment is subset specific. Nat. Immunol..

[185] Zilionis R., Engblom C., Pfirschke C., Savova V., Zemmour D., Saatcioglu H.D., Krishnan I., Maroni G., Meyerovitz C.V., Kerwin C.M. (2019). Single-Cell Transcriptomics of Human and Mouse Lung Cancers Reveals Conserved Myeloid Populations across Individuals and Species. Immunity.

[186] Morrissey M.E., Byrne R., Nulty C., McCabe N.H., Lynam-Lennon N., Butler C.T., Kennedy S., O’Toole D., Larkin J., McCormick P. (2020). The tumour microenvironment of the upper and lower gastrointestinal tract differentially influences dendritic cell maturation. BMC Cancer.

[187] Takagi S., Miyagawa S., Ichikawa E., Soeda J., Miwa S., Miyagawa Y., Iijima S., Noike T., Kobayashi A., Kawasaki S. (2004). Dendritic cells, T-cell infiltration, and Grp94 expression in cholangiocellular carcinoma. Hum. Pathol.

[188] Jinushi M., Takehara T., Tatsumi T., Kanto T., Miyagi T., Suzuki T., Kanazawa Y., Hiramatsu N., Hayashi N. (2004). Negative regulation of NK cell activities by inhibitory receptor CD94/NKG2A leads to altered NK cell-induced modulation of dendritic cell functions in chronic hepatitis C virus infection. J. Immunol..

[189] Jinushi M., Takehara T., Tatsumi T., Kanto T., Groh V., Spies T., Suzuki T., Miyagi T., Hayashi N. (2003). Autocrine/paracrine IL-15 that is required for type I IFN-mediated dendritic cell expression of MHC class I-related chain A and B is impaired in hepatitis C virus infection. J. Immunol..

[190] Martinet J., Dufeu-Duchesne T., Bruder Costa J., Larrat S., Marlu A., Leroy V., Plumas J., Aspord C. (2012). Altered functions of plasmacytoid dendritic cells and reduced cytolytic activity of natural killer cells in patients with chronic HBV infection. Gastroenterology.

[191] Kohga K., Takehara T., Tatsumi T., Ohkawa K., Miyagi T., Hiramatsu N., Kanto T., Kasugai T., Katayama K., Kato M. (2008). Serum levels of soluble major histocompatibility complex (MHC) class I-related chain A in patients with chronic liver diseases and changes during transcatheter arterial embolization for hepatocellular carcinoma. Cancer Sci..

[192] Jinushi M., Takehara T., Tatsumi T., Hiramatsu N., Sakamori R., Yamaguchi S., Hayashi N. (2005). Impairment of natural killer cell and dendritic cell functions by the soluble form of MHC class I-related chain A in advanced human hepatocellular carcinomas. J. Hepatol..

[193] Yamamoto M., Tatsumi T., Miyagi T., Tsunematsu H., Aketa H., Hosui A., Kanto T., Hiramatsu N., Hayashi N., Takehara T. (2011). alpha-Fetoprotein impairs activation of natural killer cells by inhibiting the function of dendritic cells. Clin. Exp. Immunol..

[194] Zhang S., Saha B., Kodys K., Szabo G. (2013). IFN-gamma production by human natural killer cells in response to HCV-infected hepatoma cells is dependent on accessory cells. J. Hepatol..

[195] Carbone E., Terrazzano G., Ruggiero G., Zanzi D., Ottaiano A., Manzo C., Karre K., Zappacosta S. (1999). Recognition of autologous dendritic cells by human NK cells. Eur J. Immunol..

[196] Sun C., Fu B., Gao Y., Liao X., Sun R., Tian Z., Wei H. (2012). TGF-beta1 down-regulation of NKG2D/DAP10 and 2B4/SAP expression on human NK cells contributes to HBV persistence. PLoS Pathog..

[197] Prieto J., Melero I., Sangro B. (2015). Immunol.ogical landscape and immunotherapy of hepatocellular carcinoma. Nat. Rev. Gastroenterol. Hepatol..

[198] Pinter M., Scheiner B., Peck-Radosavljevic M. (2021). Immunotherapy for advanced hepatocellular carcinoma: A focus on special subgroups. Gut.

[199] Rizvi S., Khan S.A., Hallemeier C.L., Kelley R.K., Gores G.J. (2018). Cholangiocarcinoma-evolving concepts and therapeutic strategies. Nat. Rev. Clin. Oncol..

[200] Yu M., Li Z. (2017). Natural killer cells in hepatocellular carcinoma: Current status and perspectives for future immunotherapeutic approaches. Front. Med..

[201] Zhang C., Liu Y. (2020). Targeting NK Cell Checkpoint Receptors or Molecules for Cancer Immunotherapy. Front. Immunol..

[202] Mantovani S., Oliviero B., Varchetta S., Mele D., Mondelli M.U. (2020). Natural Killer Cell Responses in Hepatocellular Carcinoma: Implications for Novel Immunotherapeutic Approaches. Cancers.

[203] Gardner A., de Mingo Pulido A., Ruffell B. (2020). Dendritic Cells and Their Role in Immunotherapy. Front. Immunol..

[204] Lurje I., Hammerich L., Tacke F. (2020). Dendritic Cell and T Cell Crosstalk in Liver Fibrogenesis and Hepatocarcinogenesis: Implications for Prevention and Therapy of Liver Cancer. Int J. Mol. Sci..

[205] Park R., Eshrat F., Al-Jumayli M., Saeed A., Saeed A. (2020). Immuno-Oncotherapeutic Approaches in Advanced Hepatocellular Carcinoma. Vaccines.

[206] Lee W.-C. (2017). Cell-mediated immunotherapy for hepatocellular carcinoma. J. Cancer Metastasis Treat..

[207] Chiang C.L., Kandalaft L.E. (2018). In vivo cancer vaccination: Which dendritic cells to target and how?. Cancer Treat. Rev..

[208] Bottcher J.P., Bonavita E., Chakravarty P., Blees H., Cabeza-Cabrerizo M., Sammicheli S., Rogers N.C., Sahai E., Zelenay S., Reis E.S.C. (2018). NK Cells Stimulate Recruitment of cDC1 into the Tumor Microenvironment Promoting Cancer Immune Control. Cell.

[209] Soares H., Waechter H., Glaichenhaus N., Mougneau E., Yagita H., Mizenina O., Dudziak D., Nussenzweig M.C., Steinman R.M. (2007). A subset of dendritic cells induces CD4+ T cells to produce IFN-gamma by an IL-12-independent but CD70-dependent mechanism in vivo. J. Exp. Med..

[210] Pampena M.B., Levy E.M. (2015). Natural killer cells as helper cells in dendritic cell cancer vaccines. Front. Immunol..

[211] Kayashima H., Toshima T., Okano S., Taketomi A., Harada N., Yamashita Y., Tomita Y., Shirabe K., Maehara Y. (2010). Intratumoral neoadjuvant immunotherapy using IL-12 and dendritic cells is an effective strategy to control recurrence of murine hepatocellular carcinoma in immunosuppressed mice. J. Immunol..

[212] Vogt A., Sievers E., Lukacs-Kornek V., Decker G., Raskopf E., Meumann N., Buning H., Sauerbruch T., Strassburg C.P., Schmidt-Wolf I.G. (2014). Improving immunotherapy of hepatocellular carcinoma (HCC) using dendritic cells (DC) engineered to express IL-12 in vivo. Liver Int..

[213] Zhou Z., Yu X., Zhang J., Tian Z., Zhang C. (2015). TLR7/8 agonists promote NK-DC cross-talk to enhance NK cell anti-tumor effects in hepatocellular carcinoma. Cancer Lett..

[214] Bray S.M., Vujanovic L., Butterfield L.H. (2011). Dendritic cell-based vaccines positively impact natural killer and regulatory T cells in hepatocellular carcinoma patients. Clin. Dev. Immunol..

[215] Morandi B., Mortara L., Chiossone L., Accolla R.S., Mingari M.C., Moretta L., Moretta A., Ferlazzo G. (2012). Dendritic cell editing by activated natural killer cells results in a more protective cancer-specific immune response. PLoS ONE.

